# Green synthesis and multifaceted applications: challenges and innovations in carbon dot nanocomposites

**DOI:** 10.1186/s11671-024-04124-3

**Published:** 2024-12-17

**Authors:** S. Varadharajan, Kirthanashri S. Vasanthan, Vidhi Mathur, N. Hariperumal, Nirmal Mazumder

**Affiliations:** 1 Manipal Academy of Higher Education, Manipal, Karnataka India; 2 Department of Civil Engineering, Manipal Institute of Technology, Manipal Institute of Technology, Manipal, Karnataka India; 3 Manipal Center for Biotherepeutics Reserach, Manipal, Karnataka India; 4 Manipal School of Life sciences, Manipal, Karnataka India

## Abstract

This paper describes the potential of carbon dot nanocomposites (CDs) synthesized from waste materials by top-down and bottom-up state-of-the-art approaches. Through sustainable practices, wastes are converted into valuable nanomaterials, solving environmental problems and pioneering advances in nanotechnology. In this paper, an overview of the synthesis aspects of CDs is presented with the formation of their versatile nanocomposites and metal/metal oxide elements. The phase of this paper has been devoted to elaborate study of the multifaceted applications of CDs in various sectors, ranging from electronics and biomedicine to environmental remediation. Although having huge potential, CDs application is presently hampered due to limitations on scalability, stability, and reproducibility. In this review paper, most profound insights have been drawn into overcoming these barriers for clear routes toward future innovations. The present research being undertaken in this area has, therefore, underscored sustainable nanotechnology to resolve global problems and achieving technological development through green synthesis. Necessitating the efficient sewage disposal systems ensuring minimum toxin generation.

**Graphical abstract:**

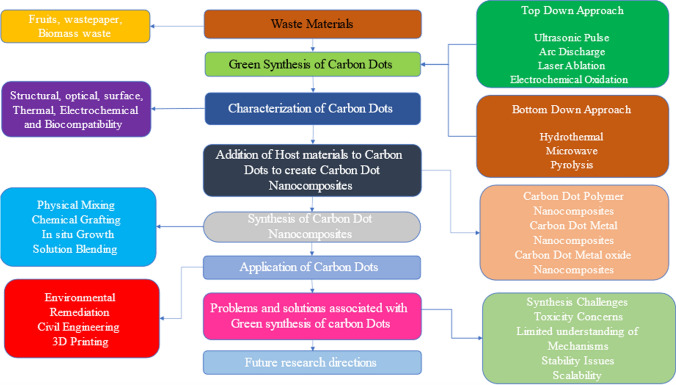

## Introduction

An enormous increase in industrialization and population has put considerable pressure on natural resources, ecosystems, and infrastructures in addition to comprehensive increase in the environmental pollution due to generation of waste material and toxins. These toxins have catastrophic impacts on environment, ecosystem and human health. Likewise, heavy metals, organic dyes, pesticides, and pharmaceuticals are well-known pollutants which have hazardous impact on living beings even causing death. Therefore, the research initiative and the regulatory measures are essential in handling and controlling hazardous impacts from such toxic chemicals [1, 2]. These issues can be effectively addressed by use of nanomaterials and zeolites. Thus, a great attention has been paid to nanomaterials due to their unique physical and chemical properties which makes it suitable for widespread application in industry, agriculture, and environmental remediation. Common nano-environmental remediation materials include metallic nanomaterials, metal oxide nanomaterials, nanomaterials based on polymers, core–shell nanomaterials, and carbon dots (CDs). CDs are commonly utilized as they exhibit nontoxicity, excellent fluorescence, optical behaviour, solubility, biocompatibility, and excellent photostability. CDs have a small diameter starting from 1 to 10 nm range [3, 4] and quasi-spherical structure of CDs show carbon atoms embedded into sp_2_ and sp_3_, with hydrophilic functional groups on their surface. Moreover, CDs have spheroidal geometry and consist of amorphous carbon with nanocrystalline regions of sp2- hybridized graphitic carbon [5, 6]. CDs can be synthesized from a wide range of carbon sources like carbon-containing organic molecules, precursors of carbon, and even from wastes. These surfaces could be functionalized with various chemical groups to provide increased functionality and stability. CDs properties can be modified by combining it with materials like polymers, metals, metal oxides, and nanoparticles to make them suitable for diverse applications. CD synthesis using toxic traditional chemical reagents is quite expensive and time consuming in addition to potential environmental hazards. Thus, there is a need to reduce dependence on toxic materials and petrochemicals. To avert this issue, CDs can be synthesised using greener approach from waste materials [7–9] like plant extracts, peanut shells, rice husk, kitchen waste, fruit or vegetable peels and microbes thereby addressing the key issues of waste management and pollution control leading to sustainable development [7–9]. Following this CDs has been synthesized with waste ionic liquid for effective sensing and detoxification of Cr(VI). Gedda et al. (2023) synthesized CDs from Azadirachta indica leaves using a one-step, one-pot sand-bath method and observed excellent antimicrobial and antioxidant properties in generated CDs [10]. Qasim et al. (2023) synthesized CDs from renewable precursors for biomedicine and environmental applications [11]. Rocco et al. (2023) conducted electrochemical synthesis of CDs from biomass waste highly preferable in sustainable applications [12]. Differing from previous approach, Bressi et al. (2022) performed large-scale synthesis of CDs using waste biomass through top down and bottom up approach , thus balancing waste management with circular economy goals [13]. Likewise, Tohamy et al. (2022) synthesized carbon quantum dots (CQDs) derived from bagasse and cellulose using microwave irradiation. The authors observed CQDs to posses excellent absorption capacity of Pb(II) ions from aqueous solution [14]. Wang et al. (2022) synthesized nanocarrier carbon dots (nCDs) using hyaluronic acid and carboxymethyl chitosan and observed nCds to exhibit good luminescence with excellent water solubility [15]. Chen et al. (2022) used forsythia-derived CDs to prepare Nylon-11 nanofibers with enhanced mechanical properties and biocompatibility [16]. Likewise, Aumber Abbas et al. (2022) prepared graphene quantum dots from biomass waste and reported suitability of generated CDs in photocatalytic, bioimaging, and sensing applications [17]. The details of synthesis of CDs from waste materials has been presented in the next section.

## Synthesis of CDs using waste materials

**Fig. 1:**
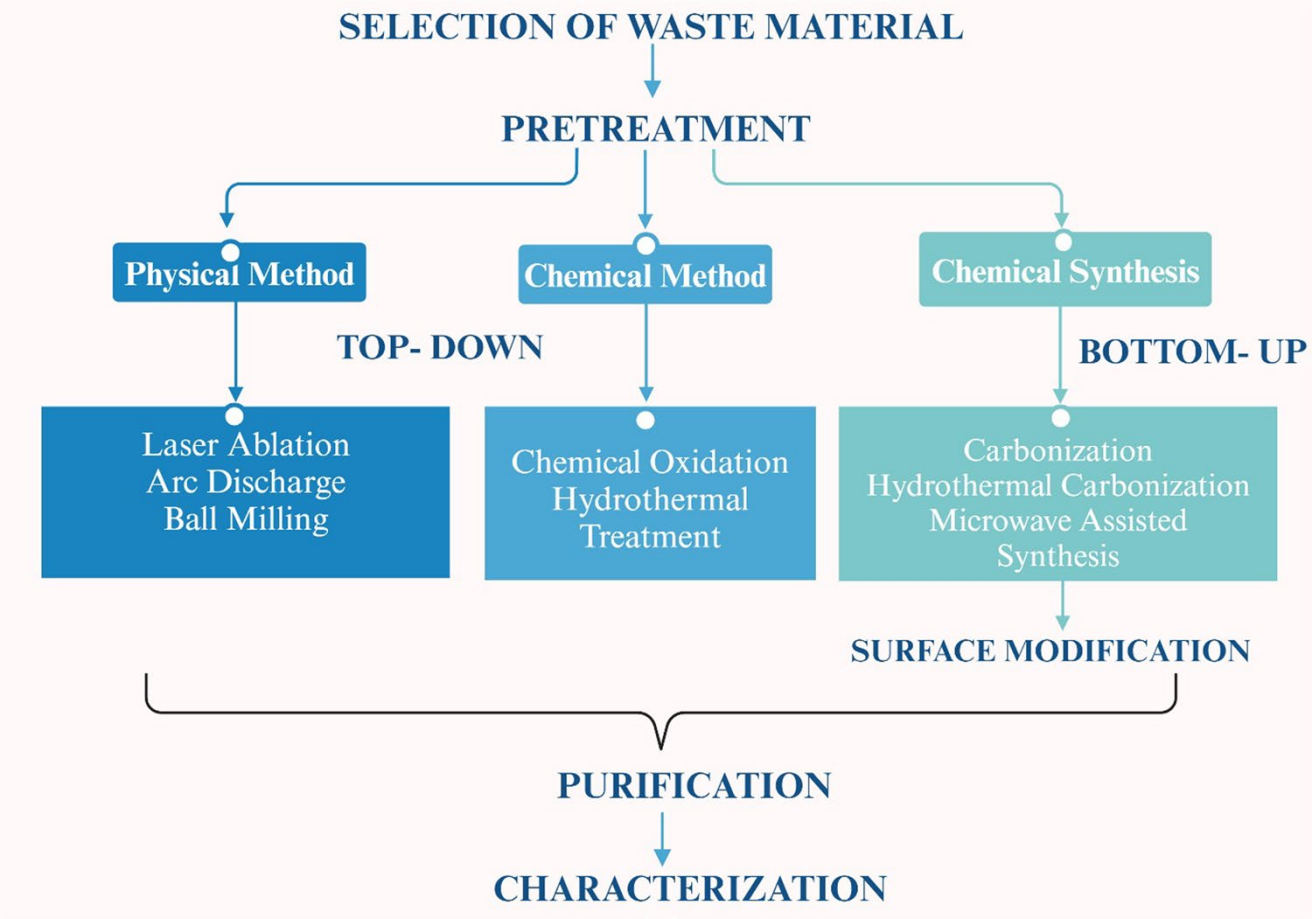
Comparison of top up and bottom up methods

**Fig. 2:**
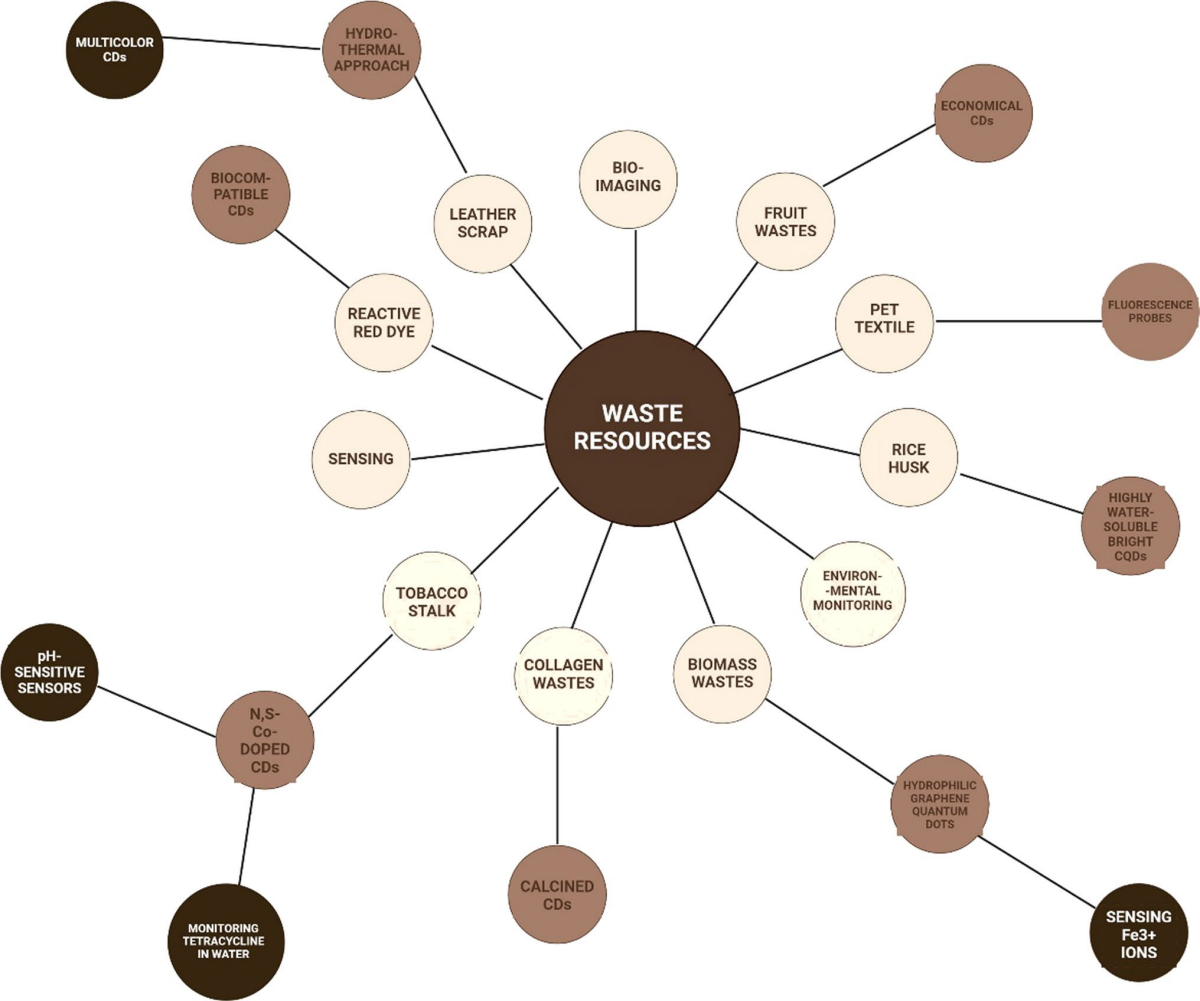
Different waste materials used for generation of CDs

The preparation of CDs from waste resources has gained wide attention and significance due to enormous environmental benefits and economy. Majority of the research works have adopted Top down & bottom up strategy for synthesis of CDs from waste materials [18, 19]. The detailed methodology and tabular comparison between different top to bottom down methods has been presented in Fig. 1 and Table 1. Using these methods, multicolour CDs with tuneable emission wavelengths were synthesized (hydrothermal/solvothermal approach) from waste leather scrap [20]. Also, materials like Rice husk straw were used to synthesize a highly water-soluble bright CQDs with excellent biocompatibility [21]. Similarly, PET textile was synthesized to form CQDs with excellent fluorescence properties which were used as fluorescence probes for detecting Fe^3+^ ions [22]. Also, collagen wastes were calcined to form solid CDs and were subsequently used in bioimaging applications [23]. In addition to above mentioned wastes, fruit wastes are gaining importance for CDs generation owing to their easy availability and economy [24–26]. Building on this trend of using agro waste in CDs generation, tobacco stalk was used for the synthesis of N, S-co-doped CDs, exhibiting enhanced photoluminescence properties. To further enhance the functionality of generated CDs, it was combined with other precursors like inorganic salts and amino acid for the fabrication of pH-sensitive sensors and tetracycline antibiotics [27, 28]. To further enhance quantum yield, CDs were synthesized from dye and solvent wastes to achieve high quantum yield and tuneable properties. Such CDs have a distinct advantage of quick water dispersion in water and good biocompatibility well suited for bioimaging application while, other biomass wastes have been significantly utilized to create a high yield and efficient hydrophilic graphene quantum dots for sensing Fe^3+^ ions. Therefore, these diversified wastes not only render a sustainable approach for CDs synthesis but also open new avenues for their other applications in the environmental monitoring, bio-imaging, and sensing applications [26, 29]. The waste materials used for synthesis for Carbon dot nanocomposites have been used shown in Fig. 2 and Table 2 and a detailed literature review pertaining to this aspect has been presented in Table 3.

**Table 1:** Top and bottom down strategy for synthesis of CDs

Aspect	Top-down strategy	Bottom-up strategy
Method	Physical or chemical breakdown of bulk materials into nanoscale	Chemical assembly from molecular or atomic precursors
Starting Materials	Carbon Rich Materials	Small molecules or monomers derived from waste materials
Process	Chemical, Mechanical and thermal treatment	Chemical process in presence of controlled conditions
Techniques Used	Laser ablation	Carbonization
	Arc discharge	Hydrothermal carbonization
	Ball milling	Microwave-assisted synthesis, Pyrolysis, Sol Gel Synthesis
	Chemical oxidation	
Purification	Filtration	Filtration
	Centrifugation	Centrifugation
	Dialysis	Dialysis
	Chromatography	Chromatography
Surface Modification	Limited to post-synthesis treatment	Easier to integrate during synthesis
Control over Properties	Less precise control over size and shape	More precise control over size, shape, and surface properties
Yield	Low	High
Scalability	Quite Challenging due to high energy consumption	Generally more scalable due to low energy consumption
Environmental Impact	Negative impact if strong oxidizing agents	Positive impact due to greener synthesis methods
Applications	High mechanical strength, electronics, photonics and sensors	Specific chemical functionality
Examples	Carbon dots synthesized from plastic waste using ball milling process	Carbon dots synthesized from biomass via hydrothermal synthesis
Advantages	Simplicity	High control over final properties
	Direct approach	Potentially more sustainable
	Less chemical waste	
Disadvantages	High energy consumption	Precision is required in controlling the reactions
	Less control over properties

**Table 2:** Waste materials used for synthesis of CDs

Waste material	Synthesis method	Applications
Agricultural Waste	Bottom Up Method (hydrothermal)	Bioimaging, Drug delivery, Environmental sensing
Food Waste	Bottom-up (pyrolysis)	Fluorescent probes, Photocatalysis, Antimicrobial agents
Plastic Waste	Bottom-up (pyrolysis)	Solar cells, Light-emitting devices, Sensors
Paper Waste	Bottom-up (microwave & hydrothermal)	Bioimaging, Biosensors, Catalysis
Sewage Sludge	Top-down (chemical oxidation)	Heavy metal detection, Photocatalysis
Coal Waste	Bottom-up (hydrothermal)	Energy storage, Supercapacitors, Photocatalysis
Fruit Peels	Bottom-up (microwave)	Bioimaging, Antioxidants, Fluorescent inks
Wood Waste	Bottom-up (hydrothermal & Pyrolysis)	Biosensors, Bioimaging, Environmental monitoring
Electronic Waste	Bottom-up (chemical vapor deposition)	Photovoltaics, LEDs, Sensors
Textile Waste	Bottom up method	Bioimaging, Antimicrobial coatings, Water purification

**Table 3:** Comparison of different methods of synthesis of CDs[214]

Method	Precursor	Size	Absorption	Emission	Plqy	Application	References
Laser Ablation	Carbon cloth in dimethylsulfoxide	3.8	276/389	448	35.40	Bioimaging ability	[215]
	Graphite target in	1–3	330	467	3.30	-	[216]
	Polyethyleneimine Graphite flakes	1.9	210/260/360	260/360	2.34	Heavy-metal-ion sensing	[217]
Electrochemical oxidation	Graphite electrode	4.0	280/380	436/438	11.20	Detection of specific ferric ions and cell imaging	[218]
	Graphite rods	6	250/300	405/565	10.00	Fluorophores for biological imaging and clinical assay application	[219]
	Ethanol	3–7	240–300	450	4.00	Fluorescence marker	[220]
Chemical Oxidation	Carbon nanoparticles	3–8	273/360/373	482	6.00	Sensitive fluorescence detection	[221]
	Carbon black	10–50	255/272/401	415	–	–	[222]
	Coke powders	–	289	410	9.20	A light-emitting diode	[223]
Sonochemical Synthesis	Cyanobacteria	1–11		300/500	–	Drug delivery	[224]
	Glucose	5	240/340	500	–	Solar energy conversion applications	[225]
	Coal	2–12	240–320	–	0.53	Environmentally friendly method	[226]
Microwave Pyrolysis	Citric acid and thiourea	5	230/340	416	30.80	Determination of Minocycline antibiotic	[227]
	Citric acid and 4,7,10-trioxa-l,13- tridecanediamine	10	210/300	440	29.00	Non-toxic bioimaging agents and solar cell	[228]
	Citric acid and Cyclen	5	350	442	27.60	Antibiotics detection	[229]

## Mechanisms of CDs synthesis

### Top-down methods

**Fig. 3:**
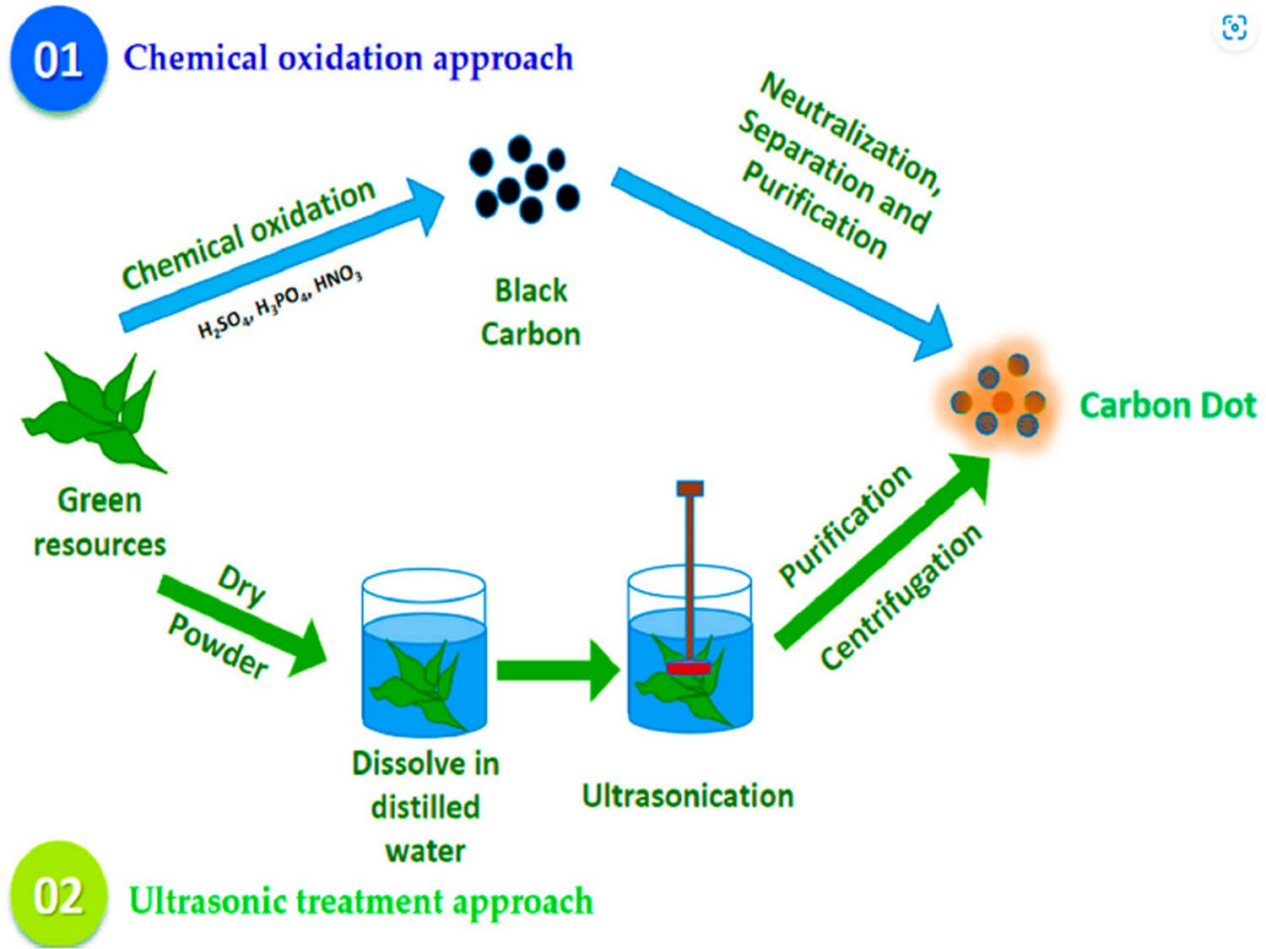
Top down approach for synthesis of CDs (ultrasonic pulse velocity and chemical oxidation approach) [8]

*Ultrasonic pulse method* Ultrasonic pulse method is one of the forms of top-down approach which reduces larger than carbon molecules into smaller carbon dot particles using high energy ultrasound waves. This technique is mainly adopted due to uniform impact, eco friendliness, low cost, scalability, precise control over size, shape and other surface properties [30] as shown in Fig. 3. Jing et al synthesized CDs from cigarettes using ultrasonic sound waves. The synthesized CDs was functionalized using polyethylene oxide to exhibit green fluorescent under UV light [8]. However, non uniform heating is a key challenge faced in the ultrasonic method. It could attribute to localized thermal effects of ultrasonic waves which negatively impacts the reaction efficiency in comparison to microwave or direct heating method. These challenges need to be effectively addressed through further research in optimization of CDs synthesis through ultrasonic techniques. Ultrasonic technique involves sound waves with frequency greater than the audible range (> 20 kHz). Among the different ultrasonic approaches, the direct method involves synthesizing of CDs through carbon precursors. This process is initiated through preparation of a solution through use of carbon sources like carbohydrates, polymers and organic molecules [31]. Thereafter, these solutions are subjected to the ultrasonic sound waves through a probe immersed in the solution. The ultrasonic waves cause cavitation where tiny bubbles form and collapse in the liquid. This collapse creates localized pressure and temperature leading to carbonization and fragmentation of precursors. The resulting carbon atoms are subjected to quenching and cooling effects of the surrounding liquid due to which they reassemble to form CDs. The direct ultrasonic method is highly advantageous due to high yield, tuneable optical properties and rapid synthesis of CDs [32, 33]. Nevertheless, this method may involve hazardous and toxic precursors which results in stability and reproducibility issue. As alternative, Ultrasonic method have better control over synthesis process which facilitates incorporation of dolphins and additional functional groups increasing stability and reproducibility but in direct synthesis, methods are more costly and time consuming [34].

*Arc-discharge* Arc discharge method is one of the top-down approaches for synthesis of CDs and which involves generation of an electric arc between two graphite electrodes in an inert atmosphere comprising of argon or helium [35]. A plasma created by the high energy arc causes vaporization and subsequent cooling of the graphite electrodes leading to CDs formation. This process causes fragmentation of carbon structures to nanoscale dots due to very high temperatures in the arc zone. CDs generated through this method elucidate a high crystalline behaviour due to rapid cooling of the crystals which can be controlled by tuning parameters like arc pressure, arc cooling and electrode composition. This technique produces CDs with well-defined graphite structures and relatively narrow sized distribution makes them highly preferable for robust thermal stability and high electrical conductivity [36]. However, this method can induce defects on CDs surface which can be detrimental or beneficial depending upon the type of application.

**Fig. 4:**
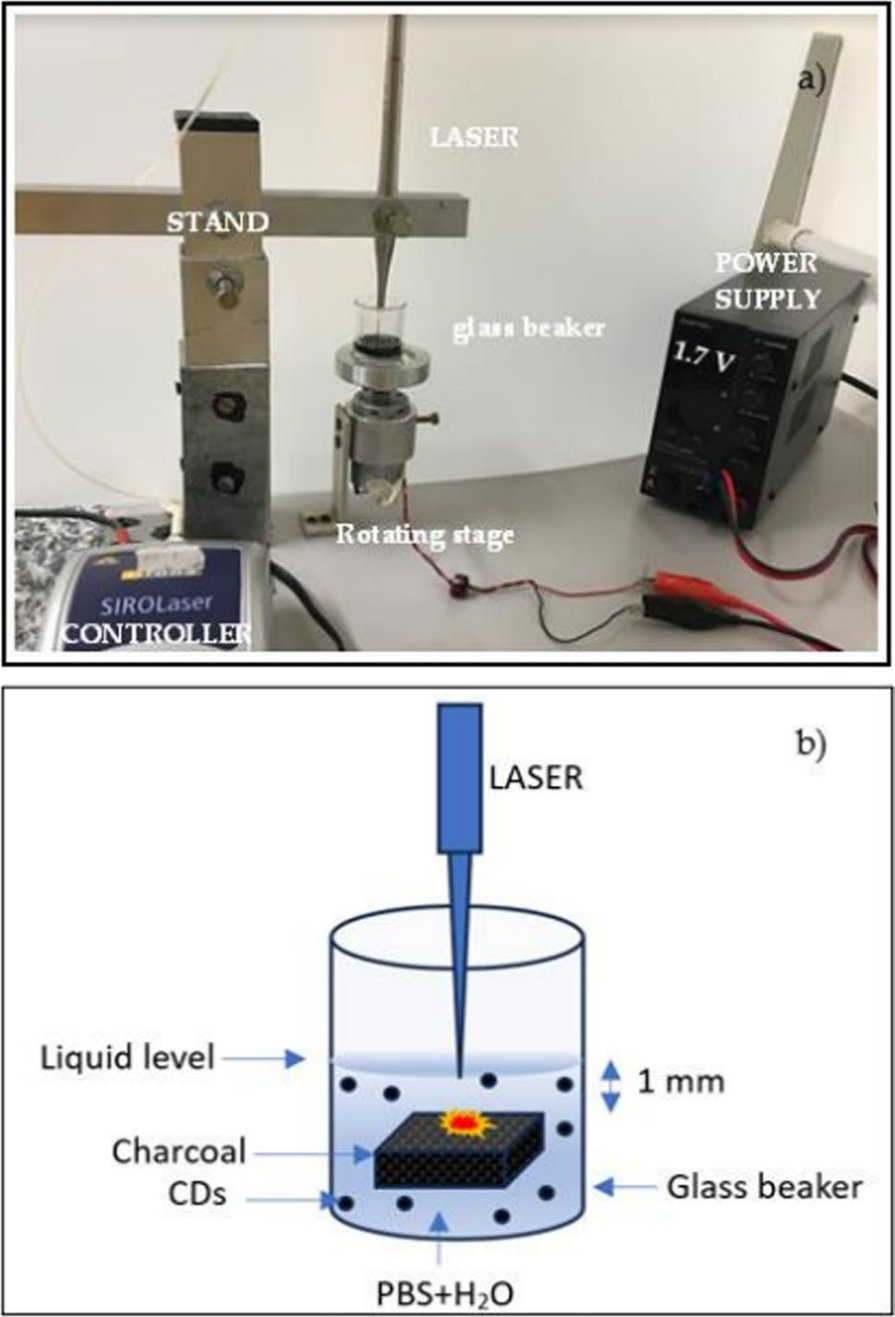
Generation of CDs by laser ablation [199]

*Laser ablation* Laser ablation is a versatile and efficient method for synthesizing CDs^37^,^38^, leveraging the high-energy pulses of a laser to ablate a carbon-rich target material submerged in a liquid medium (Fig. 4). This process involves directing laser pulses onto the target, which results in the rapid heating and subsequent vaporization of the material's surface. The vaporized material then cools and nucleates into CDs within the liquid medium. The mechanism behind this process primarily involves the fragmentation of carbon bonds due to the intense laser energy, leading to the formation of small carbon clusters. These clusters undergo further structural rearrangement, stabilizing into spherical nanodots with characteristic photoluminescent properties. The synthesized CDs are highly valued for their unique optical properties, including strong fluorescence, which makes them particularly useful in bioimaging, biosensing, and optoelectronic applications. Additionally, due to their biocompatibility and low toxicity, CDs produced via laser ablation are being explored for use in drug delivery systems^39^,^40^and as contrast agents in medical diagnostics.

**Fig. 5:**
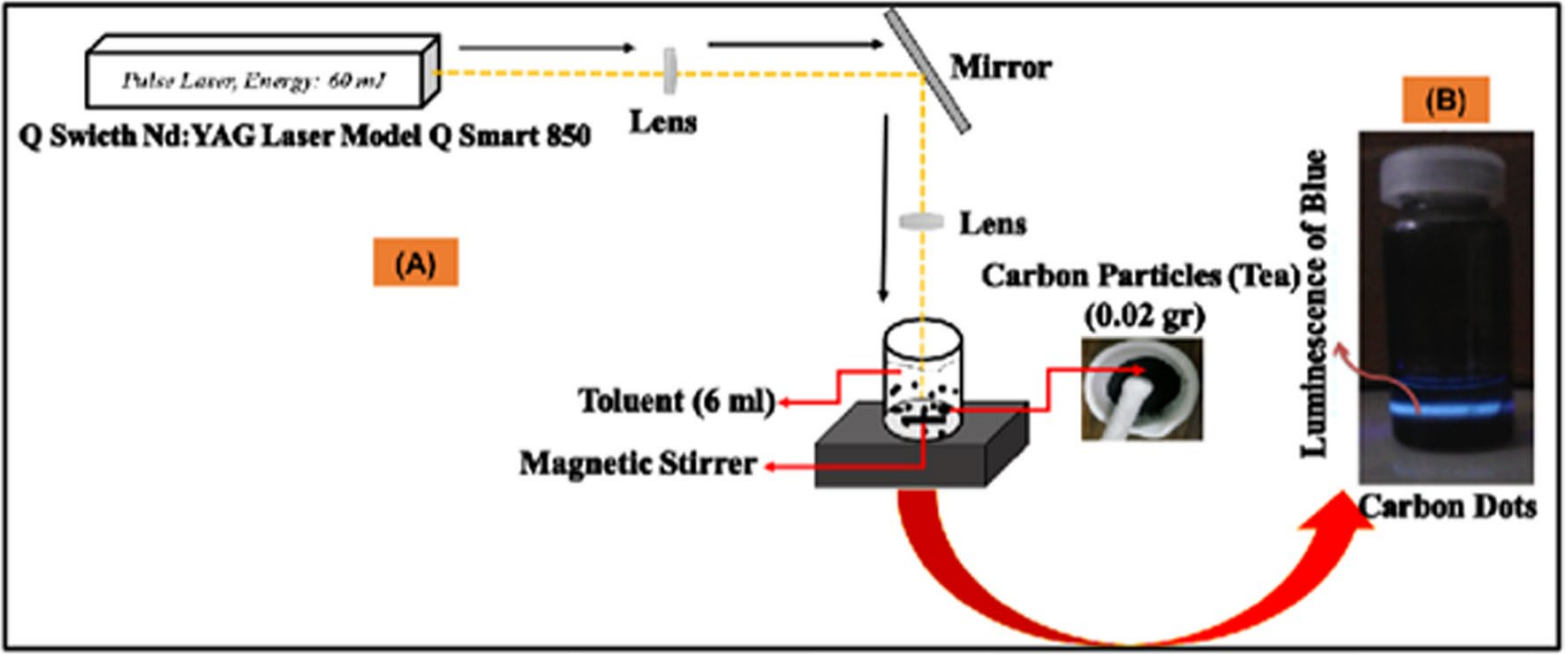
(**A**) Generation of CDs by laser ablation and (**B**) Luminance of CDs [200]

**Fig. 6:**
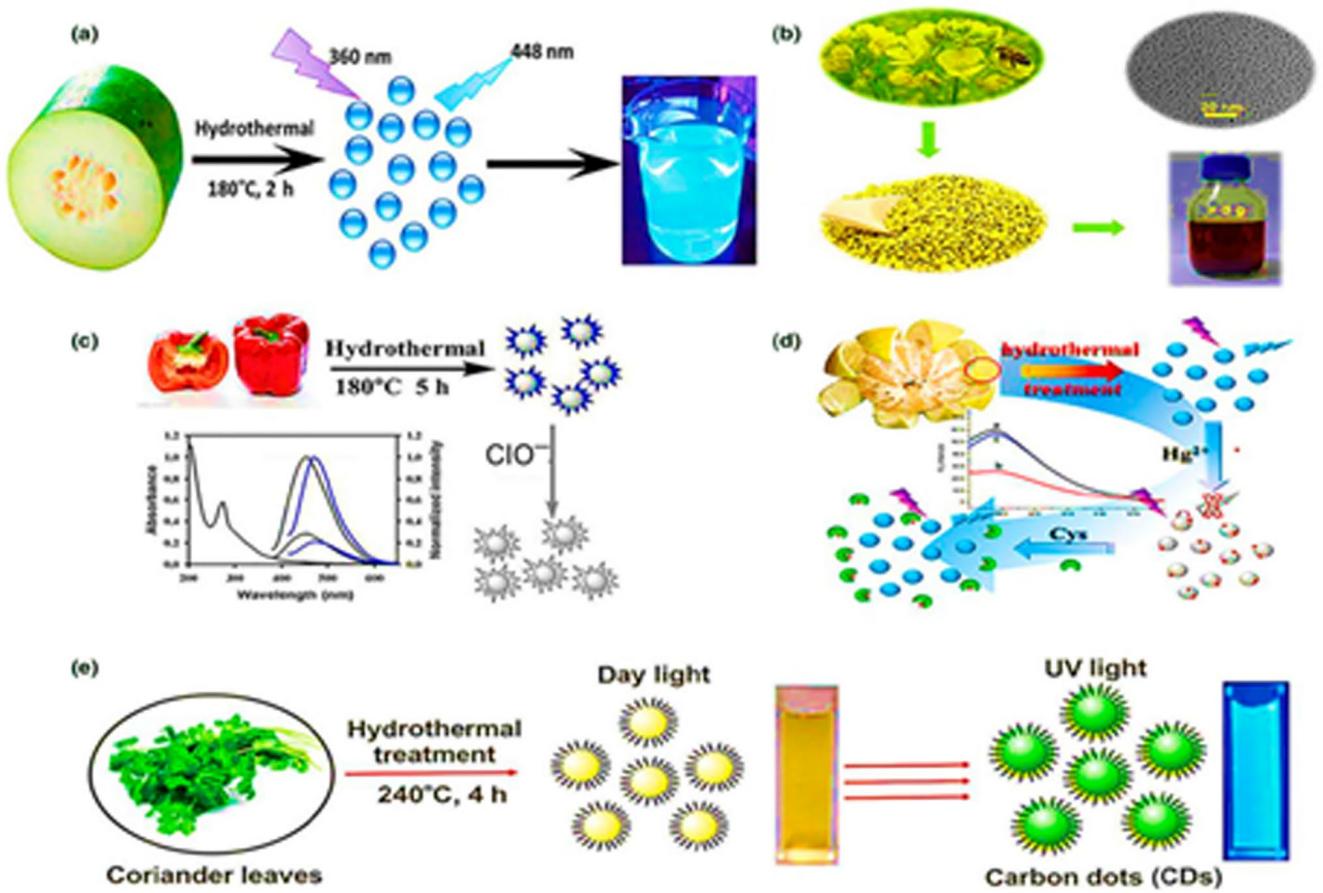
Preparation of biomass carbon Dots (BCDs) by means of hydrothermal methods from **a** Winter melon^201^, **b** bee pollens^202^, **c** Sweet pepper^203^, **d** Pomelo Peel^204^ and **e** Coriander leaves 205[9, ]

- Laser ablation is a synthesis method that involves utilizing a laser beam to disintegrate a carbon source into nanomaterials, as depicted in Fig. 4. This technique is straightforward and efficient, requiring minimal use of chemicals, which enhances its appeal for environmentally conscious applications. The interaction time between the laser beam and the material is crucial in determining the quality of the resulting structures [41]. Typically, carbon nanodots (CDs) are synthesized using laser ablation at a wavelength of 1064 nm. For example, CDs have been synthesized by directing the laser beam onto carbon-rich materials, such as tea-derived CDs, suspended in a colloidal solution of toluene for a duration of three hours, as shown in Fig. 5. The resulting CDs were then employed as fluorescent materials for bioimaging applications. A significant advantage of laser ablation is its ability to control the incorporation of functional groups without the need for hazardous solvents, offering a clean and efficient approach that minimizes chemical usage [41]. However, a notable limitation of this method is the relatively low quantum yield, which ranges between 4.5 and 18% [42].
- *Electrochemical oxidation* As illustrated in Fig. 6, the electrochemical approach is a versatile method for synthesizing carbon quantum dots (CQDs) with precise control over their optical properties and sizes. This method generally follows a top-down strategy, where larger carbon-based materials, such as graphite electrodes, are fragmented under an applied electrical potential to produce high-quality CQDs [43]. The electrochemical approach is highly efficient, allowing for the execution of heterogeneous redox reactions. It enables precise control of the potential difference between two electrodes and the monitoring of the current flowing through the electrolytic cell, which provides valuable insights into the properties of the resulting CQDs. Moreover, the use of electrons as redox agents is advantageous due to their low cost, non-polluting nature, and ease of application, making them "green" reagents in chemical synthesis. CQDs produced via this method are typically rich in functional groups, which enhances their applicability in sensor technologies. The electrochemical approach offers several benefits, including high production yield, low cost, straightforward size control, and high purity of the synthesized CQDs [44, 45]. However, the primary drawback of this method is the longer reaction time required for synthesis.

### Bottom-up methods

**Fig. 7:**
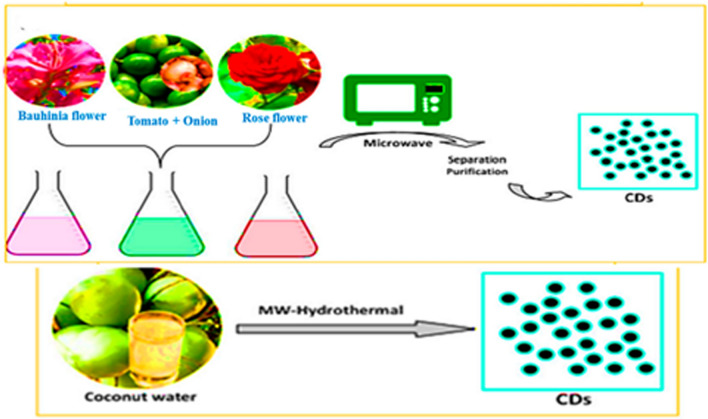
Microwave method for CDs synthesis [206]

- *Hydrothermal/Solvothermal Synthesis* Hydrothermal and solvothermal synthesis are widely used bottom-up methods for producing carbon dots, utilizing organic precursors in a sealed, high-pressure environment. In hydrothermal synthesis, the reaction takes place in water, while solvothermal synthesis uses other solvents, such as ethanol or ethylene glycol. The organic precursors, which can include small molecules, polymers, or biomass, are subjected to elevated temperatures (typically 150–250 °C) and pressures inside an autoclave. Under these conditions, the organic materials undergo carbonization, breaking down into smaller fragments that nucleate and grow into carbon dots (Fig. 6).
- The precursor choice, reaction conditions (time, temperature and pressure), solvent and precursor have a significant influence on shape, size, surface chemistry and optical properties of CDs. This method has an inherent advantages like simplicity, wide range of functional groups, scalability, customized properties and Eco friendliness. The CDs produced from these methods elucidated strong photo luminance highly preferable in optoelectronics, sensors and bioimaging. In addition, this process is environment friendly as it requires minimum amount of chemicals since it uses natural precursors like plant extracts or waste materials. CDs derived from citric acid and ethyledeamine subjected to a high temperature of 190 °C for 2 h showed a quantum yield of 58% in aqueous solutions [46]. Hydrothermal method is considered to be one of the most eco-friendly processes among other methods of synthesis of CDs since it uses water as solvent. The authors boiled the precursors at high temperature 120–240 °C with reaction time varying from 3 to 12 h to create a hope friendly CDs [47]. Moreover, hydrothermal process is often used in large scale synthesis as tuning properties of CDs is very easy and precursors from waste resources can be used for synthesis of CDs. The solvothermal method is similar to hydrothermal method but ethanol is used as solvent in solvothermal method in place of water. The choice of solvent during synthesis is governed by quantum yield fluorescence optical, optical property and purity of CDs. For example, Chen et al. synthesized trichromatic CDs using solvothermal method using single precursor, 2,5-dimethylbenzenesulfonic acid, by varying the solvents, such as NMP, DMF, and formamide, while keeping other conditions constant. As elucidated by Fig. 7, solvent governed both quantum yield and mechanical properties of CDs. But this method has an inherent disadvantage of higher energy consumption and increased reaction time.
- Figure 6 shows different types of bio-mass being used as the precursor for generation of CDs. The sources of materials for green synthesis of CDs vary from Agapanthus africanus leaves 48 to biowastes [49] (Fig. 6e). The generated CDs exhibited excellent dispersion varying from 1 to 5 nm with uniform size distribution (20–40 nm). Recently, Khalifa  et al. used bee pollen as precursor for synthesizing CDs. The authors derived 3 g CDs from 10 g bee pollen (Fig. 6b). High quality CDs were synthesized using up conversion fluorescence sweet pepper as the precursor through hydrothermal treatment at 180 °C in an autoclave [50] as shown in Fig. 6c. The generated CDs showed a size distribution from 2 to 7 nm [51] along with the carbon quantum yield of 19.3% %. However, CDs derived from sweet pepper and cabbage resulted in narrower quantum yield [52, 53]. Specifically, from sweet pepper had a particle size varying from 2 to 4 nm with quantum yield of 6.9% (Fig. 6d). In contrast, CDs generated from orange juice demonstrated high quantum yield of 26% [54, 55] with a particle size of 2.5 nm. Meng et al. [57] synthesise CDs from citrus lemon juice in NaOH solution resulted in 12.1% quantum yield.

*Microwave-assisted synthesis* The microwave synthesis method is a bottom-up approach where CDs precursors are combined with materials such as Bauhinia flowers, tomatoes, onions, and rose flowers to produce nitrogen-doped carbon dots (N-CDs) in an aqueous solution, as illustrated in Fig. 7. This technique operates at significantly lower temperatures compared to the hydrothermal process. Similarly, Rai et al. [58] reported the microwave-assisted synthesis of fluorescent carbon dots (FCDs) using sulphur containing lignosulfonate lignin as the carbon source, where sulphur served as a doping agent. The synthesized CDs, which featured a narrow particle size distribution, demonstrated excellent stability and fluorescence properties, making them suitable for drug loading and release applications. The variety of functional groups present on the surface of lignin-derived CDs contributes to their stability and enhanced fluorescence characteristics.

**Fig. 8:**
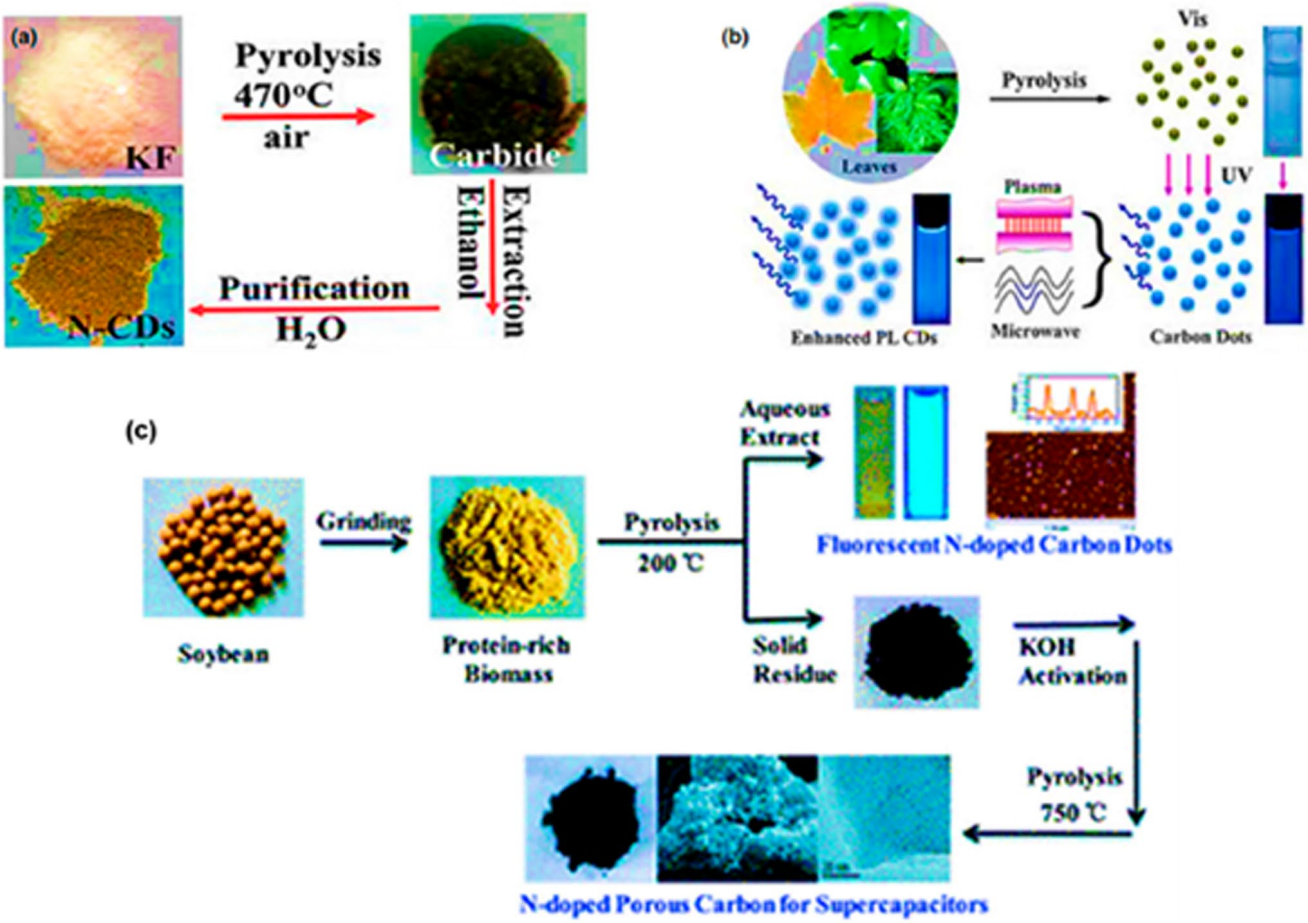
Different pyrolysis synthesis methods of carbon dots **a** Konjac flour, (230**b** Plant leaves 231) **c** Soya beans [208]

**Fig. 9:**
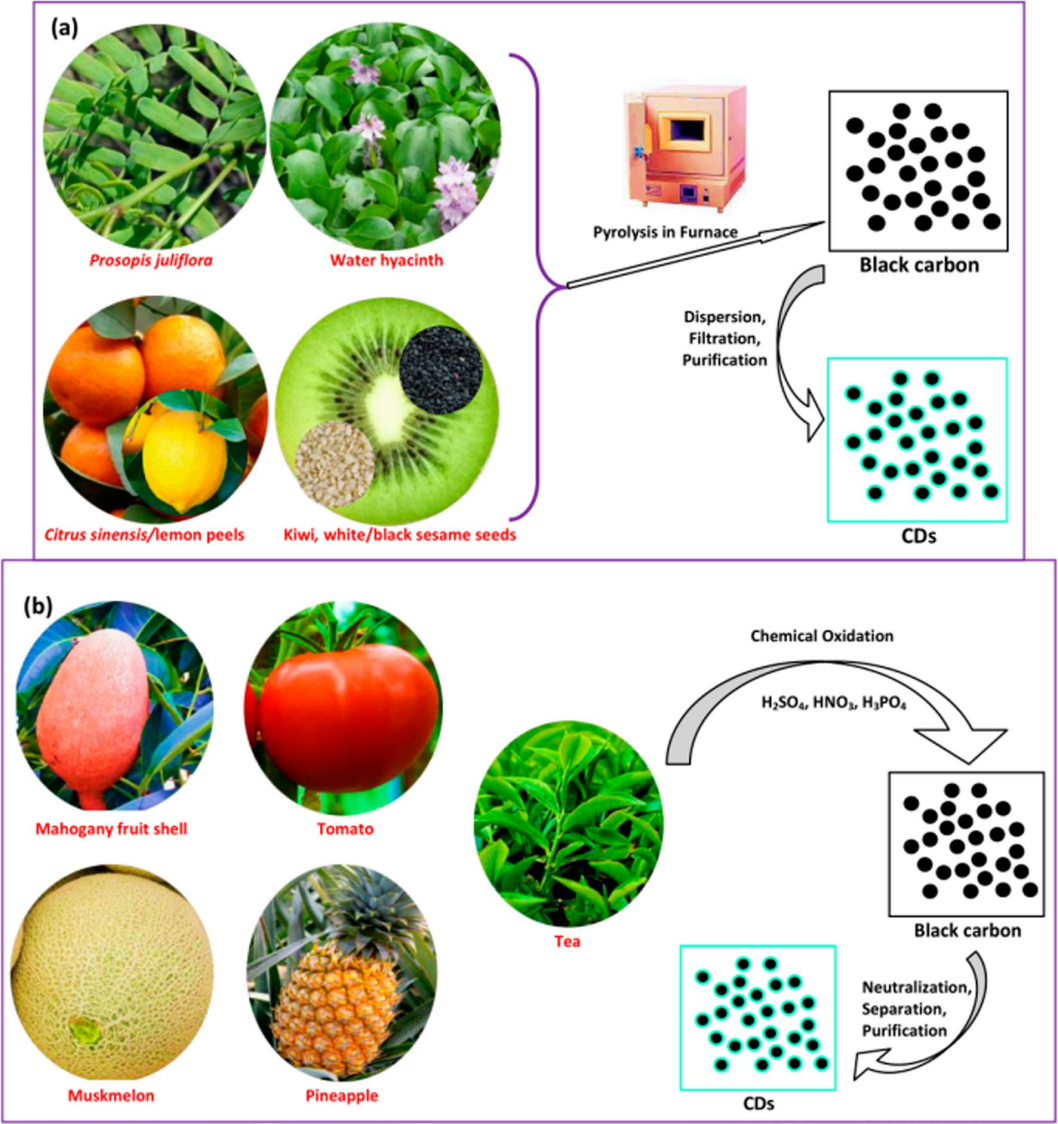
Pyrolysis process [206]

*Pyrolysis* The pyrolysis process was first introduced by Xu et al. [59] in widely adopted for synthesis of CDs. Speight 60[] described carbonization as thermal decomposition of materials rich in carbon in inert atmosphere yielding (especially natural resources) yielding carbonaceous residues (Fig. 8a, b). This method has numerous advantages like cost economy, rapid reaction time, scalability, simplicity and cost-effective nature. However, aggregation tendency during carbonization is one of the biggest challenges faced in the pyrolysis process. To resolve this issue, a novel two step carbonization process was developed which combined simultaneous extraction of CDs (nitrogen doped) and porous carbon (Fig. 8c). The process initiates with low temperature carbonization (200 °C) of soybean material which results in formation of fluorescent carbon dots thereby preserving the atoms (N, S, O) and microstructure of the raw material. The first step is followed by carbonization at high temperature (750 °C) with KOH as catalyst which causes partial graphitization of pre carbonation residue. This results in a carbon with porous structure with sequential network of mesopores and micropores resulting in a high specific surface area of 1663.1 m^2^/kg (Fig. 8c). The pyrolysis process is also elucidated in Fig. 9.

## Characterization properties of CDs

### Structural characterization

- *Transmission Electron Microscopy (TEM):* This technique is used to visualize the size, shape, and distribution of CDs within the composite. High-resolution TEM can provide insights into lattice structure and crystallinity.
- *X-Ray Diffraction (XRD):* This method provides information on the crystalline nature and phase purity of the CDs and composites. The broadening of peaks indicates the amorphous or semi-crystalline nature typical of carbon dots.
- *Raman Spectroscopy:* It is used to assess the graphitic or amorphous nature of the CDs by analysing the D and G bands. This technique also provides information on defects and the degree of *sp*^2^ hybridization.

### Optical characterization

- *UV Absorption Spectroscopy:* This method analyses the absorption behaviour of CDs, typically showing strong absorption in the UV region due to π–π* transitions. The absorption peak positions can indicate the size and surface states.
- *Photoluminescence (PL) Spectroscopy:* This method evaluates the emission properties of CDs, which typically exhibit excitation-dependent emission due to different emissive traps. PL is crucial for understanding the electronic structure and surface passivation.
- *Time-Resolved Photoluminescence (TRPL):* This technique measures the fluorescence lifetime, providing insights into the recombination dynamics of excitons and the influence of surface defects or dopants.

### Surface characterization

- *Fourier Transform Infrared Spectroscopy (FTIR):* This method identifies functional groups on the surface of CDs, which are critical for determining the surface chemistry and potential interactions within composites.
- *X-Ray Photoelectron Spectroscopy (XPS):* The XPS provides elemental composition and chemical state information, particularly valuable for understanding the surface functionalities and bonding environment of the CDs.
- *Zeta Potential Measurement:* This measurement assesses the surface charge of CDs in suspension, providing insights into their colloidal stability and interactions with other components in a composite.

## Thermal and mechanical characterization

- *Thermogravimetric Analysis (TGA):* This analysis evaluates the thermal stability and composition of CDs by monitoring weight loss as a function of temperature. This can indicate the presence of different phases or the decomposition temperature of the composite.
- *Differential Scanning Calorimetry (DSC):* The DSC measures the thermal transitions such as glass transition, crystallization, and melting of CDs, providing insights into their thermal properties.
- *Mechanical Testing:* Techniques like tensile testing or nanoindentation assess the mechanical properties (e.g., Young’s modulus, hardness) of CDs, particularly when the dots are incorporated into polymer matrices.

### Application-relevant characterization

Depending on the intended application (e.g., energy storage, catalysis, biomedical), additional specific characterization techniques might be required:

- *Electrochemical Characterization (for energy applications):* Techniques like cyclic voltammetry (CV), electrochemical impedance spectroscopy (EIS), and galvanostatic charge–discharge (GCD) are used to evaluate the electrochemical performance of CDs.
- *Biocompatibility Studies (for biomedical applications):* Cell viability assays, hemocompatibility tests, and in vivo studies are necessary to assess the safety and effectiveness of CDs in biological environments.

This detailed comparison between different characterization methods of CDs has been shown in Table 4.

**Table 4:** Detailed comparison of different characterization methods for CDs

Characterization method	Purpose	Information provided	Advantages	Limitations
Transmission Electron Microscopy (TEM)	Structural visualization of CDs	Size, shape, distribution, crystallinity (lattice fringes)	High resolution, detailed structural info	Requires complex sample preparation, expensive, not ideal for bulk analysis
X-Ray Diffraction (XRD)	Crystallinity and phase identification	Crystalline vs. amorphous nature, phase purity	Fast, provides bulk information	Limited sensitivity for amorphous materials, requires crystalline sample for peak detection
Raman Spectroscopy	Graphitic/amorphous nature, structural defects	D and G band intensity, degree of disorder (ID/IG ratio)	Non-destructive, useful for defect analysis	Difficult to interpret complex structures, may require complementary techniques
UV–Vis Absorption Spectroscopy	Optical properties	Absorption peaks (UV region), π–π* transitions, size, surface states	Simple, fast, quantitative	Limited structural information, no spatial resolution
Photoluminescence (PL) Spectroscopy	Emission properties	Excitation-dependent emission, surface passivation, emissive traps	Sensitive to defects and surface states	Fluorescence quenching may complicate interpretation, excitation-dependent behavior can be complex
Time-Resolved Photoluminescence (TRPL)	Fluorescence lifetime measurement	Recombination dynamics, influence of defects	Provides detailed recombination data	Requires specialized equipment, may be time-consuming
Fourier Transform Infrared Spectroscopy (FTIR)	Surface chemistry analysis	Identification of functional groups (e.g., OH, COOH)	Non-destructive, simple preparation	Limited to surface groups, may require complementary techniques for full surface analysis
X-Ray Photoelectron Spectroscopy (XPS)	Elemental composition, chemical state	Surface elemental analysis, bonding environment	Sensitive to surface chemistry, quantitative	Expensive, requires high vacuum, only surface analysis (top ~ 10 nm)
Zeta Potential Measurement	Colloidal stability and surface charge	Surface charge, stability in suspension	Simple, rapid, useful for predicting interactions in suspensions	Only useful for colloidal systems, limited structural information
Thermogravimetric Analysis (TGA)	Thermal stability and composition analysis	Decomposition temperature, phases, weight loss	Provides detailed thermal behavior	Only bulk analysis, cannot resolve structural details
Differential Scanning Calorimetry (DSC)	Thermal transitions	Glass transition, crystallization, melting points	Provides detailed thermal property data	Limited structural information, requires large amounts of material
Mechanical Testing (Tensile/Nanoindentation)	Mechanical properties assessment	Young’s modulus, hardness, tensile strength	Quantitative mechanical data	Bulk mechanical data may not be representative of nanomaterial behavior
Cyclic Voltammetry (CV)	Electrochemical behavior	Redox activity, specific capacitance	Fast, effective for energy-related applications	Limited to electrochemical analysis, requires conductive samples
Electrochemical Impedance Spectroscopy (EIS)	Ion diffusion and resistance	Resistance, charge transfer, ion mobility	Provides comprehensive electrochemical properties	Requires specialized equipment, limited to conductive materials
Biocompatibility Studies	Assessment of safety in biological systems	Cell viability, hemocompatibility, toxicity	Critical for biomedical applications	Requires biological assays, time-consuming

### Literature review pertaining to characterization of CDs

Characterization techniques are essential for understanding and detailed analysis of CDs [61]. The characterization study enables researchers to examine different aspects related to CDs like structure, size, shape, surface chemistry and morphology [62]. A detailed understanding of these aspects is required to tune CDs properties for enhancing their application in diverse fields like biomedicine, optoelectronics, energy storage and sensing [63]. The structure of CDs shows variation depending upon synthesis methods and chemical structure (amino acids, oxygen are polymer based etc.). Thus, multiple characterization techniques have often been used for determination of chemical structure of CDs [64]. CDs elucidate exceptional optical and electrochemical properties making them highly preferable in fields like bioimaging, biosensing and therapeutic development making them an ideal choice for gene delivery, photothermal therapy and drug delivery [65].

**Fig. 10:**
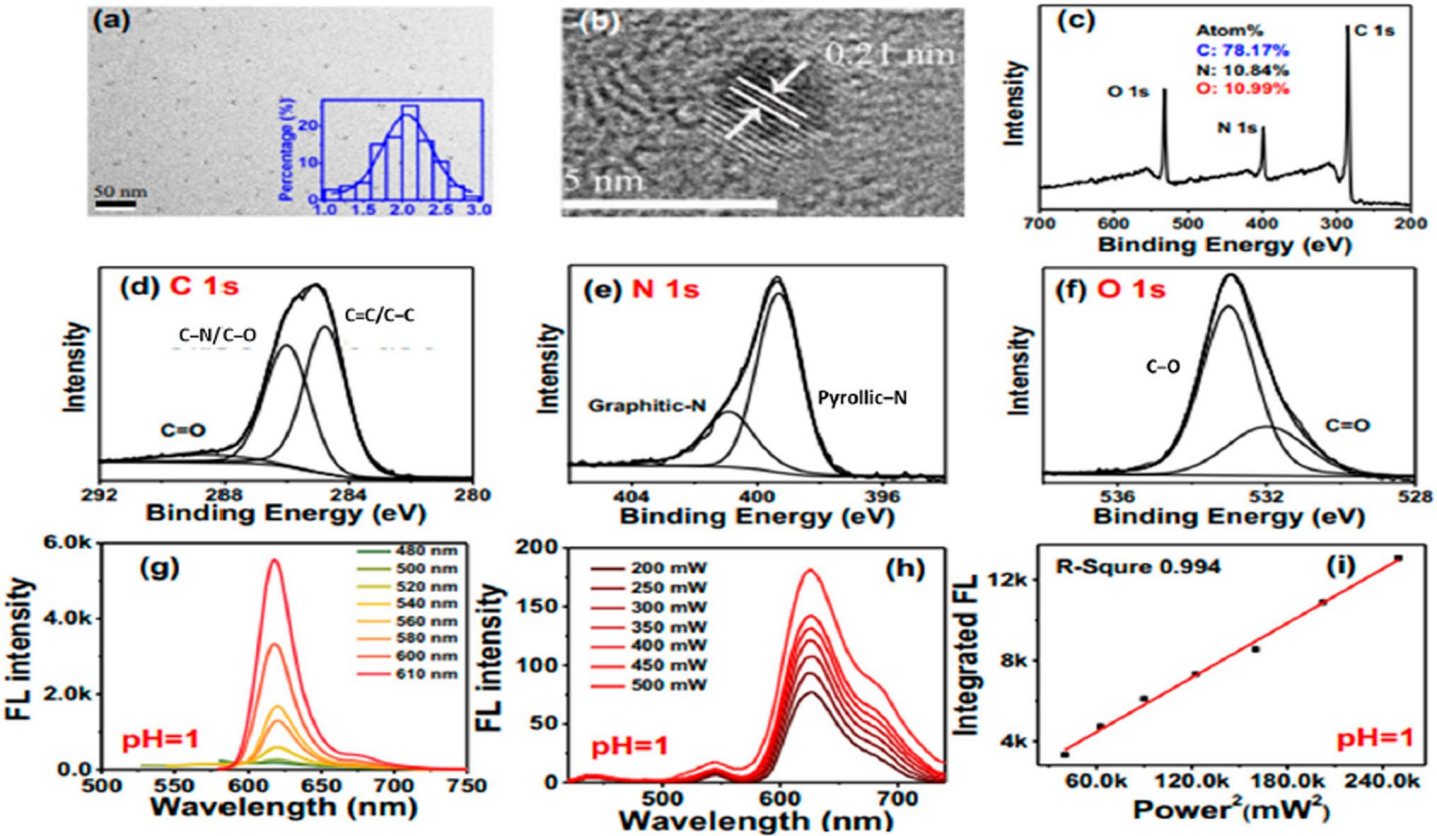
Structure, composition, and optical properties of protonated CDs. **a** TEM image of CDs (inset, particle size distribution of CDs); **b** high-resolution TEM image of CDs; **c**–**f** full scale XPSspectra, high-resolution C 1*s*, N 1*s*, and O 1*s* XPS spectra of CDs; **g**, **h** one-photon and two-photonfluorescence (FL) spectra of protonated CDs in deionized water; **i** relationship of two-photon FLintensity and femtosecond (fs) laser power [209]

Transmission electron microscopy (TEM) was used to take images of CDs at a resolution of 50 nm. The Authors observed uniform distribution of CDs in aqueous solutions with average particle size of 2 nm as elucidated in Fig. 10a.

Figure 10b shows alignment of CDs at (1,0,0) graphite plane with lattice spaced at 0.21 nm. Moreover, the distribution pattern of carbon atoms in CDs considerably effects the interlayer spacing. For example, CDs has a close resemblance to structure of graphite and may have an interlayer distance of 0.21 nm for (1,0,0) lattice plane similar to graphene. Conversely, the interlayer distance can increase due to presence of defects or an amorphous structure. The increase in degree of carbonization tend to increase the graphitic structure resulting in close interlayer distance. Moreover, interlayer distance is significantly influenced by choice of precursors, with different precursors resulting in CDs with diverse interlayer distances.

The X-ray photoelectron spectroscopy (XPS) analysis of the CDs, is elucidated in Fig. 10c. From Fig. 10c, atomic ratios of 10.99% for oxygen, 10.84% for Nitrogen and 78.17% for carbon can be observed. The high resolution XPS spectra of CDs is shown from Fig. 10d to 10 f from which presence of following components can be seen, (a) *sp*^2^ C=C carbon (284.76 eV), (b) *sp*^3^ C−N/C−O carbon (286.0 eV), and C=O groups (288.6 eV) in the C 1*s* spectra. The N 1*s* spectra is shown in Fig. 10e shows peaks of graphitic nitrogen (400.9 eV) and pyrrolic nitrogen (399.3 eV). Also, while the O 1*s* spectra (Fig. 10f) show peaks of 533.0 eV and 531.9 eV for groups C−O−C/C−O−H and C–O. The other details are elucidated from Fig. 10g–i.

**Fig. 11:**
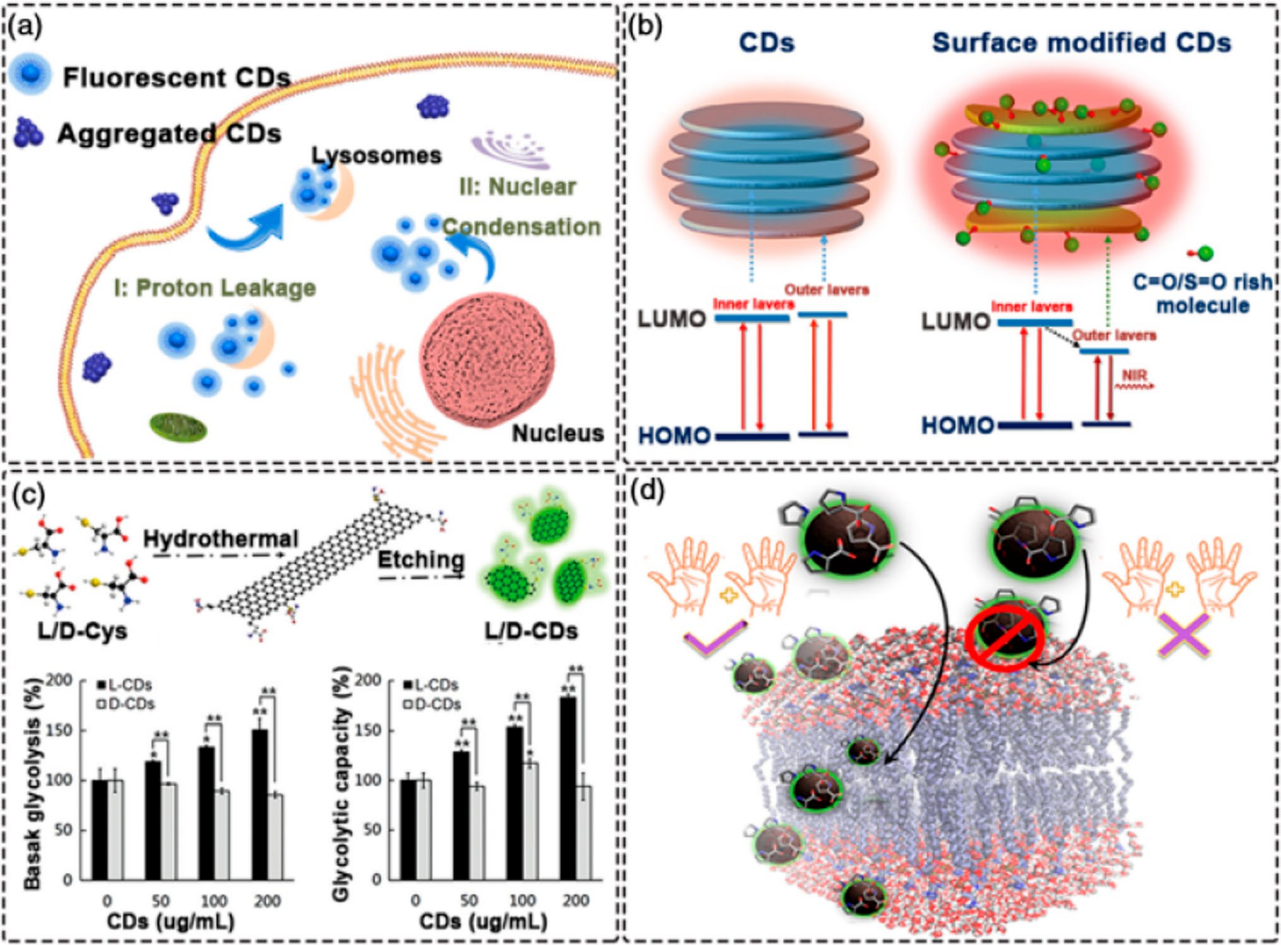
Optical properties of CDs. **a** High quantum yield CDs for visualization of lysosomes [210]. **b** NIR-emitting CDs for bio-imaging [211]. **c** Chiral CDs for the treatment of T24 cells [212]. **d** Chiral CDs demonstrating high selectivity for cell membranes [213]

CDs generally exhibit excellent optical absorption in the UV and visible spectrum range. Irrespective of the synthesis method, the majority of the CDs have an absorption band between 260 and 323 nm [66]. However, absorption Spectra of CDs depend upon type of bonds namely, (a) Pi–Pi transition of C–C bonds, (b) n-Pi transition of C–O bonds. Nevertheless. Surface passivation of CDs different molecules changes their absorption properties by shifting them towards longer wavelengths. The recent research works have shown great interest in optical properties of CDs including photo Luminance, high fluorescence quantum yields, Red/Near Infra-Red emission and in NIR driven applications (Fig. 11a). In biological and biomedical applications, CDs are used for photothermal heating and chiral luminescence. High Quantum Yield are advantageous for in vivo tracking of CDs. Due to the limited penetration of short-wavelength light in biological tissues, most CDs absorb in the UV range and emit blue-green light, making detection challenging. This limitation can be partially addressed by using blue-green emission CDs with Quantum Yield. CDs' acidophilic properties allow for passive visualization of lysosomes in living cells. For example, green-emitting CDs with a 90.49% Quantum Yield were produced using branched polyethyleneimine (bPEI) and rose Bengal (RB) as precursors. As shown in Fig. 11b, there is growing interest in Red/Near Infra-Red emitting CDs due to their low autofluorescence interference, deep tissue penetration, and minimal tissue absorption. These CDs have been explored for in vitro and in vivo biological system observations. Red and Near Infra-Red -CDs have been used in cell imaging to label cancer cells, where they primarily aggregate in the cytoplasm and cell membrane. Our lab successfully synthesized red-emitting CDs with high Quantum Yield using o-phenylenediamine as a precursor [67, 68]. These CDs were employed as fluorescent probes for in vitro and in vivo imaging, enabling bioimaging. Moreover, the resulting L-CDs are suitable for long-term in situ imaging of the Golgi body cells due to their high light stability and biocompatibility. The dynamic changes of the Golgi body cells during early viral infection stages can be observed using these CDs. Additionally, chiral cysteine has been extensively used as a chiral ligand and stabilizer to modify nanomaterial properties. When L (or D)-cysteine-derived chiral CDs were tested on human bladder cancer T24 cells, L-CDs up-regulated glycolysis, whereas D-CDs did not have the same effect (Fig. 11c). For example, D-proline, with inverted chirality, preferentially interacts with liposome mimics or the cell membrane (Fig. 11d).

Excitation-dependent photoluminescence (PL), or excitation-dependent fluorescence emission, is one of the most notable characteristics of carbon quantum dots (CQDs). Generally, the PL of CQDs is dependent on the emission wavelength and intensity [69]. Zhang's et al. studied the emission behaviour of CQDs when exposed to 470 nm light at different concentrations [70]. They observed that as the excitation wavelength and CD concentration increased, the PL intensity of yellow-emitting CQDs initially peaked at a maximum λex and then decreased. This study demonstrated that CDs exhibit similar excitation-dependent photoluminescence activity in comparison to other luminescent CDs [71]. Similarly, by heating o-phenylenediamine under KCl catalysis, Ding, H. et al. [72] produced a series of CDs with tuneable PL emissions ranging from 442 to 621 nm, FQYs of 23–56%, and production yields of 34–72%. Extensive analysis revealed that the variations in these CDs' optical characteristics are determined by differences in graphitization extent, graphitic nitrogen content, and oxygen-containing functional groups. These variations can be controlled by managing the deamination and dehydrogenation processes during synthesis. The appropriate CDs are dispersed into polyvinyl alcohol (PVA) to create blue, green, yellow, and red emissive films and LEDs. Red, green, and blue emissive CDs are also mixed to produce various warm, standard, and cool white LEDs (WLEDs) with a high colour rendering index (CRI) [36].

## Applications of CDs

**Fig. 12:**
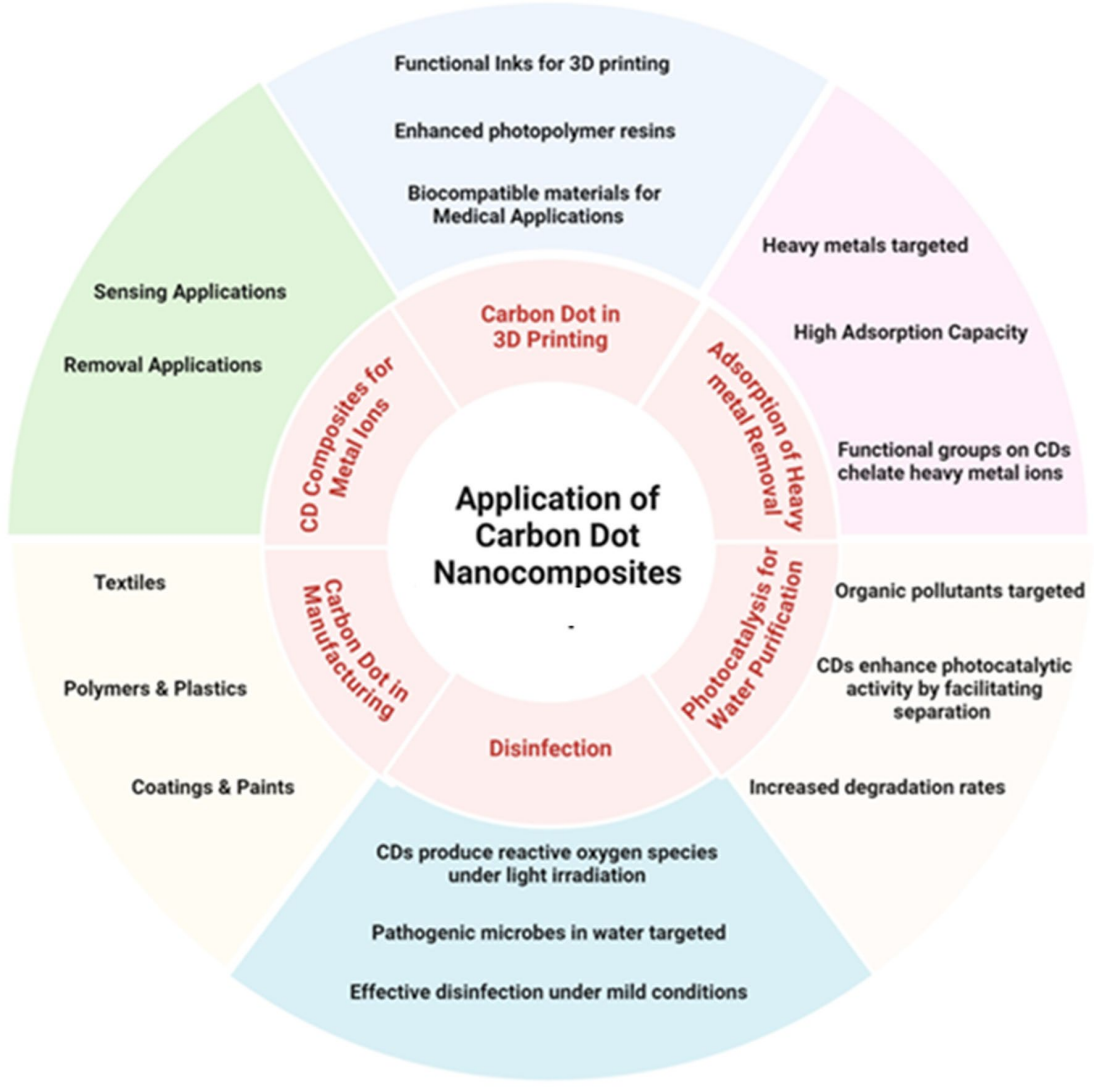
Applications of Carbon dot nanocomposites

**Fig. 13:**
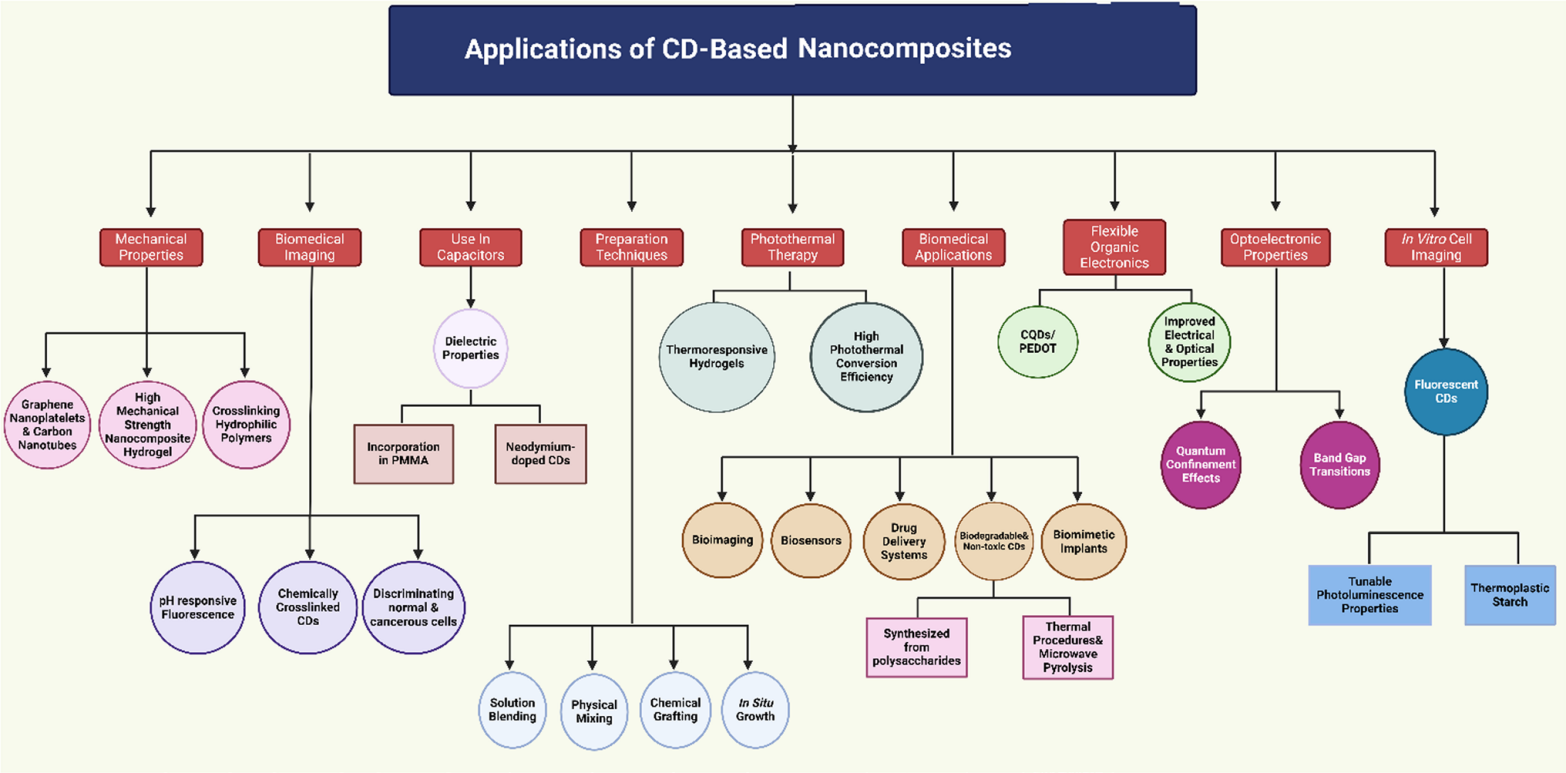
Application of CD based nanocomposites based on functional properties

**Fig. 14:**
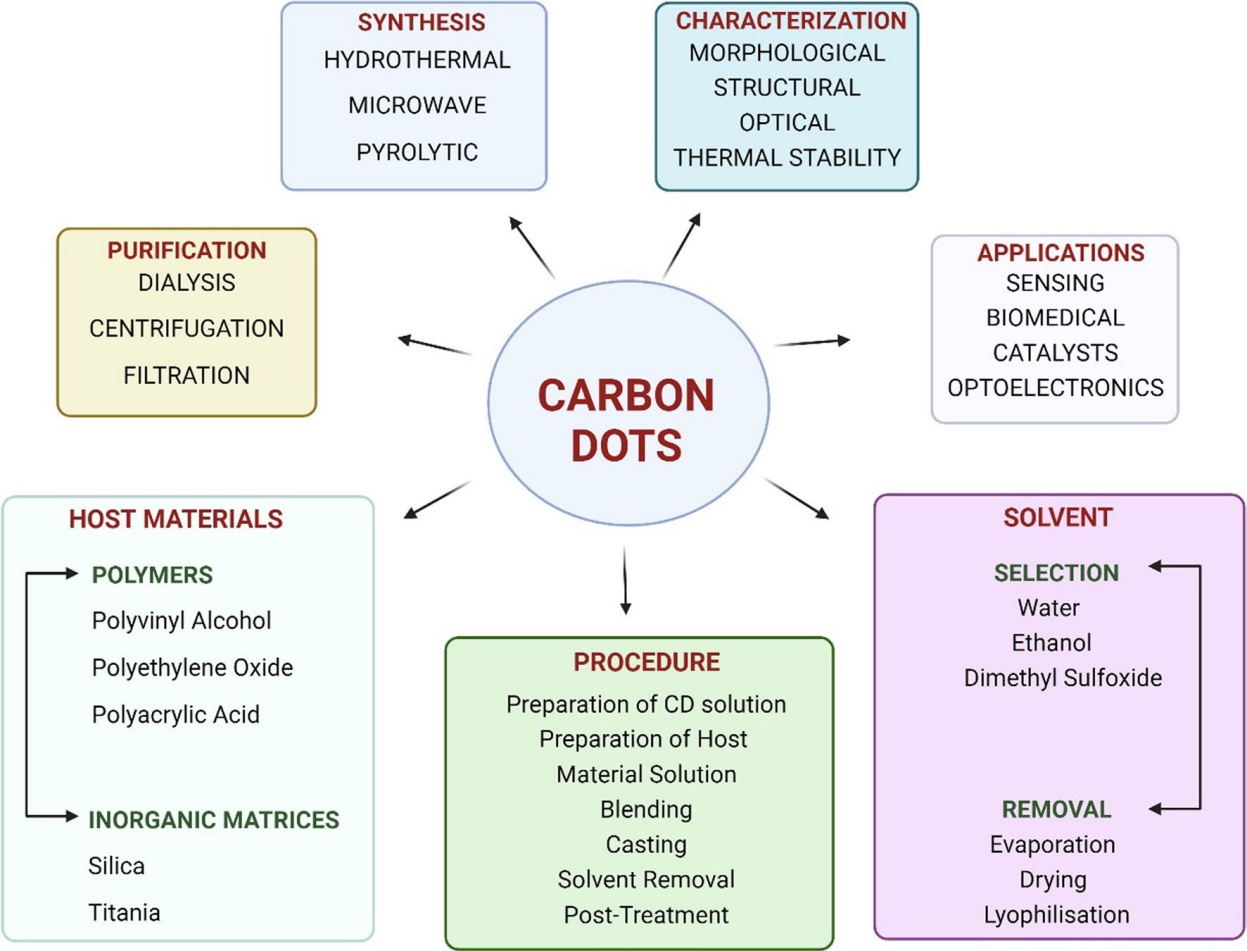
Different aspects of Carbon Dot Nanocomposites

The CDs has variety of applications (Figs. 12, 13 and 14, Tables 5 and 6) as described in detail in the sub sections below.

**Table 5:** Role of CDs in different applications

Application sector	Specific use cases	Role of CDs and CDs nanocomposites	Advantages	Challenges
Environmental Remediation	Water purification, removal of heavy metals, degradation of organic pollutants	Catalysts in photocatalytic degradation, adsorbents for heavy metals, fluorescent sensors for pollutant detection	High efficiency in removing contaminants, renewable, non-toxic	Scaling up for industrial applications, potential regeneration of adsorbents
Civil Engineering	Enhancement of concrete durability, self-sensing materials, anti-corrosive coatings	Reinforcing agents in concrete composites, sensors for structural health monitoring	Improved durability, cost-effective sensing solutions, potential for real-time monitoring	Long-term stability in harsh conditions, large-scale application challenges
Reducing Environmental Pollution	Air and water purification, carbon capture, pollutant degradation	Photocatalysts for degrading harmful pollutants, fluorescent markers for pollution tracking	Sustainable and effective in degrading various pollutants, cost-effective	Efficiency under real-world conditions, long-term stability
3D Printing	Incorporation of CDs into resins for enhanced mechanical properties, biocompatible 3D-printed structures	Additive in printing materials for enhanced strength, functionality, and biocompatibility	Enhanced mechanical properties, increased biocompatibility for medical applications	Compatibility with various resins, potential toxicity concerns in biomedical uses
Manufacturing Sector	Improved thermal and mechanical properties of composites, development of sensors for monitoring manufacturing processes	Nanofillers for mechanical strength improvement, use in developing smart sensors for process monitoring	Enhanced material properties, development of real-time sensing technologies, cost-effective manufacturing processes	Integration with existing manufacturing processes, cost of carbon dot production at scale

**Table 6:** Potential industrial applications of CDs nanocomposites

Industry	Application	Description
Optoelectronics and Displays	Light-emitting devices (LEDs)	Used as efficient emitters in LEDs with high luminescence and tunable emission properties
Optoelectronics and Displays	Photodetectors and Solar Cells	Enhances the efficiency of solar cells and photodetectors by absorbing a wide light spectrum
Biomedical Applications	Bioimaging and Biosensing	Allows tracking and detection of cells or molecules in bioimaging and detecting pathogens in biosensors
Biomedical Applications	Drug Delivery and Therapy	CDs used in targeted drug delivery systems and photothermal therapy for cancer treatment
Environmental Monitoring and Remediation	Sensing of Environmental Pollutants	Detects heavy metals, toxic gases, and organic pollutants for environmental monitoring
Environmental Monitoring and Remediation	Wastewater Treatment	CDs act as photocatalysts to degrade organic pollutants in wastewater
Energy Storage and Conversion	Supercapacitors and Batteries	Enhances energy storage performance in supercapacitors and batteries
Energy Storage and Conversion	Fuel Cells	Improves the efficiency of hydrogen production and oxygen reduction reactions in fuel cells
Sensors and Actuators	Chemical Sensors	Detects gases, ions, or chemicals by fluorescence quenching or enhancement properties
Sensors and Actuators	Temperature and Pressure Sensors	Changes optical properties under varying conditions, useful in environmental sensing
Catalysis	Photocatalysis	Efficient electron transfer under light irradiation for reactions like hydrogen evolution
Catalysis	Enzyme Mimics (Nanozymes)	Mimics enzymes to catalyze biological and chemical reactions, useful in industrial processes
Textile and Coating Industries	Antibacterial Coatings	Provides antimicrobial properties for textiles or surfaces, useful in medical applications
Textile and Coating Industries	Functionalized Textiles	CDs embedded in fabrics offer UV resistance, fluorescence, and self-cleaning capabilities
Agriculture	Pesticide Detection and Removal	Detects pesticides in soil or water, helping agricultural monitoring
Agriculture	Plant Growth Stimulation	Enhances plant growth by improving photosynthetic activity
3D Printing and Additive Manufacturing	Nanocomposites for Functional Printing	CDs incorporated into polymer matrices for printing functional smart devices and sensors
Cosmetics and Personal Care	UV Protection and Skin Care	CDs block UV rays and are non-toxic, making them suitable for skin care formulations

### Environmental remediation application

The increasing global population and industrialization have significantly polluted water sources, adversely affecting ecosystems and human health. CDs have grabbed researchers' attention for pollutant detection and remediation due to their unique fluorescent properties, high biocompatibility, low environmental impact, and abundance of functional groups. The focus towards CD/metal and CD/metal-oxide nanocomposites has grown because composite materials allow better stability, recyclability, portability. CDs and CQDs (carbon quantum dots) are considered of great interest for environmental remediation, principally due to their excellent properties, like high catalytic activity, biocompatibility, and excellent electron transfer capability. These materials have been well investigated for their ability in the photocatalytic degradation of pollutants [73, 74], such as dyes, antibiotics, and conversion of CO_2_. For example, CDs have been used as photosensitizers to enhance the photocatalytic degradation of tetracycline without the requirement of external photosensitizers or co-catalysts due to efficient use of photoinduced electron transfer (PET) process more efficiently.The BiOBr/Bi_2_WO_6_ hybrid materials based on CQDs showed good photocatalytic performance for water pollutant decomposition due to enhancement of visible light absorption and high electron-transfer properties in the Bi_2_WO_6_ matrix. The photocatalytic activity can be further enhanced by functionalization or doping CDs^75^,^76^, ^77^, like red luminescent CQDs of g-C_3_N_4_ used to enhance CO_2_ reduction. CDs have found application in the degradation of dyes, thus offering a green and efficient solution for the treatment of water. The other applications of CDs are in the treatment of air pollution, the development of sensors, and photocatalysts for the detection and removal of gaseous pollutants [78, 79]. Also, CDs composites enable the most efficient use of the solar spectrum for the degradation of organic pollutants. However, ecotoxicity aspect of these nanomaterials needs to be investigated. In summary, the development of CD-based photocatalysts represents a sustainable and practical approach to treat a variety of environmental issues, from water and air pollution to energy shortage [79] as depicted in Fig. 12 and Table 5.

### CDs in civil engineering

Addition of nano particles to CDs enhance their properties making them highly suitable for civil engineering applications (Figs. 12, 13 and 14). More often, CDs are used as an additive in cement-based composites enhancing their properties. This could be attributed to the fact that CDs have a small size (in range of few nanometres), water-soluble property and presence of hydrophilic groups which assist in micro filling of pores in cement matrix thereby increasing density of interfacial transition zone leading to increment of mechanical strength. Authors observed a 16.8% compressive strength increment of cement-based composites at 28 days, with a minimal dosage of 0.08 wt%. [80]. CDs nanocomposites can also act as structural regulators, chemical modifiers, and reinforcing agents, leading to good water dispersibility, enhanced mechanical properties, increased durability highly suitable for efficient construction. Moreover, the eco-friendly and cost-effective characteristics of CDs [81, 82] make them appropriate for up-scaling industrial applications which justifies use of costly carbon nanomaterials like graphene. The mechanical improvements of CDs, along with their luminescent properties [83] makes them highly preferable in structural health monitoring. CDs are very popular in sensing and contaminant removal applications leading to sustainable construction practices [78, 84]. Furthermore, the integration of CDs into Luminescent Solar Concentrator (LSCs) induces energy efficiency in buildings resulting in self-sustainable urban structures. Therefore, multifunctional capability of CDs exhibits their transformational impact on civil engineering applications like induction of Carbon dots (CDs) in concrete may increase strength due to several reinforcement mechanisms. At first, the small size of CDs leads to micro-filling of voids in cementitious matrix enhancing the strength and density of the interfacial transition zone. Also, CDs act as nucleation sites thereby promoting dense hydration products. Moreover, presence of the hydrophilic groups like carboxyl and amide groups on the surface of CDs adsorb the cement particles thereby enhancing the strength of cementitious matrix [80, 85]. Nevertheless, carboxyl-premodifier carbon nano tube (CNTs) and carbon nanofiber (CNFs) in concrete leads to better compressive strength due to better dispersion in the concrete matrix. The use of carbon fibres in ultra-high-performance concrete (UHPC) has also shown improved interfacial bonding [86, 87], mainly when carbon fibres are pretreated to introduce oxygen-containing groups that develop strong Van der Waals force and hydrogen bonds withing the cement matrix. Also, applying carbon textile reinforcement without surface modification (such as nano-silica deposition) contributes to a significant increase in bond strength and pullout resistance in concrete. Thus, CDs nanomaterial combinations [88, 89, 90] with other materials  including   polypropylene microfibers, shows a synergistic effect while incorporated into the concrete mix and boosting the engineering properties. Therefore, it can be concluded that use of CDs in concrete leads to enhanced strength and sustainability advocating their use in construction applications. CDs, in this way, can be used as a promising pathway to improve the mechanical properties of concrete by enhancing the dispersion, interfacial bonding, and increased formation of hydration products.

### CDs in reducing environmental pollution

CDs have emerged as one of the potential solutions for reducing pollution of the environment, owing to their high-water solubility, good biocompatibility, tuneable fluorescence, and low toxicity (Figs. 12, 13 and 14). In particular, CDs are potent sensors for detecting and eliminating heavy metals, organic contaminants and pesticides [91,93,92]. In particular, the photoluminescence and chemical stability of the CDs based sensors are highly suitable in detecting Pb^2+^ and Cr^6+^ in drinking water for ensuring safety. Meanwhile, CDs exhibit excellent electron transfer and optical characteristics due to which they can be used in the photocatalytic decomposition of organic pollutants, dyes, and antibiotics. Additionally, integration of graphite quantum dots and carbon nitride (g-C_3_N_4_ QDs) with CDs strongly enhance their photocatalytic and adsorption capacity highly preferable for the treatment of wastewater contaminants like methyl orange and bisphenol A. Furthermore, CDs can be used as fluorescence probes for detecting and removing the pollutants like tetracycline through photoinduced electron transfer process. The green synthesis of CDs (from biomass and other sustainable sources) with nanoparticle addition renders economic and environmental benefits advocating their applications in environmental remediation [94, 95]. In addition, CDs have been explored in removing air pollutants because of their tuneable properties and high electron-transfer capacities which is quite useful for sensing and removal of gaseous pollutants. All these properties of CDs make them versatile in their overall multifunctionality including sensing, photocatalysis and adsorption, making them effective in the mitigation of environmental contamination.

### CDs in manufacturing sector

CDs have been in the spotlight of the manufacturing industry lately due to their high photoluminescence, chemical stability, and biocompatibility. Such nanocomposites combine CDs with, polymers, metals, and ceramics to improve their material properties making them suitable for wide range of applications (Figs. 12, 13 and 14). For instance, CDs have often been used in electronic display technologies like quantum dot LEDs, which can provide considerably more brightness, colour accuracy, and energy efficiency in comparison to traditional display systems. Additionally, owing to their high conductivity, they also show applications in printed electronics, sensors, and energy storage devices promising excellent potential in future electronic components. In the field of material science, CDs are being used for adhesive^96^  and coating [97] applications to enhance mechanical strength, thermal stability [98], and environmental resistance [78]. CDs use reduces structural weight and enhances durability which makes them potent for automotive and aerospace industry.

Application of CDs in additive manufacturing and 3D printing^99^,^100^, paves the way for producing high-resolution and multifunctional products tailored for particular applications. CDs properties can be easily tuned by surface functionalization and doping which extend their applicability to the development of smart materials^102^,^101^ requiring high optical, electrical, and mechanical characteristics . Therefore, CDs are drivers of innovation across the manufacturing sector and offer an efficient and sustainable solution to overcome contemporary challenges of the industrial sector.

CDs offer both improved printing precision and additional functionality of materials in 3D printing, e.g., of hydrogels and resins [103, 104], by enhancing light absorption and with present photoluminescence and electric conductivity capabilities. CDs also improve the mechanical strength, UV mitigation and water resistance properties of food packaging materials [105, 106] thereby extending the shelf life of food products due to their antimicrobial and antioxidant properties. Recent interest in CDs application is due to their ability to form non-covalent bonds with wide range of molecules and polymers which makes them suitable for sensing, bioimaging, and therapeutic applications. CDs represent a class of solution-processed, non-toxic, inexpensive, and stable laser material, which in combination is required for obtaining high-quality imaging and holographic displays. CDs displays semiconductor like properties thus they are used in various photocatalytic applications[107,108 reference], environmental cleaning, and organic synthesis thereby promoting green manufacturing initiative. The development of CDs has also found applications in molecular electronics, optical sensing probes in combination with logic gates and memory elements advocating their use in advanced manufacturing technologies. Moreover, their strong versatility, ease of preparation from waste resources further promotes the potential of CDs in sustainable manufacturing. CDs perform a vital function in the domain of energy conversion [109, 110] and storage like light-emitting diodes, photovoltaic cells, and supercapacitors for the realization of an effective manufacturing system with reduced energy wastage.

### CDs applications in 3D printing

In the field of 3D printing, CDs have emerged as an exciting class of nanomaterials with unique beneficial properties for different applications (Fig. 12). They are capable of fluorescence thus multi-colour polyvinyl alcohol CDs fluorescent film was used to enhance the printing quality of inkjet printer. The CDs can contain the liquid metal nanodroplets in them to enhance the colloidal stability [111, 112] and the photoluminescence of the 3D/4D-printed objects, which can respond to the stimuli of both water and laser irradiation, thereby expanding the area of stimuli-responsive functional materials. Generally, the CDs are synthesized directly within the polydimethylsiloxane elastomer base to attain self-luminescent devices. These devices possess stable and enhanced fluorescence resulting and can be effectively used in the anti-counterfeit devices. Furthermore, CDs enables ultrafast visible-light-induced polymerization in additive manufacturing with high monomer conversion resulting in 3D printed hydrogels with excellent photoluminescent properties. Likewise, incorporating CDs with PLA-based filament enhances cellular adhesion and growth [113, 114] making them preferable for bone generation and monitoring of healing process through bioimaging. CDs derived from natural plant dyes are also highly luminescent and eco-friendly and may find further opportunities in safe printing systems. Incorporation of CDs into these 3D-printed sensors can enhance their electromechanical properties and long-term stability increasing their potential in a wide variety of sensing applications with carbon-based nanomaterials, including graphene and carbon nanotubes. CDs are well suited for high-sensitivity and selective sensing applications (sensing of the Fe^3+^ ion, biological imagining, and anti-counterfeiting). CDs are also integrated into flexible capacitive sensors which are integral elements of Internet of Things (IOT) ecosystem. Therefore, CDs in 3DP science opens a window for creating truly advanced multifunctional, responsive, and high-performance materials. Generally, CDs improve 3D printing technology because of improvement in functional properties of printed materials. CDs can be incorporated into different polymer matrices, providing enhanced photoluminescence applicable to strengthen applications like anti-counterfeiting and bioimaging.

For example, CDs incorporated within polydimethylsiloxane elastomers help to create complex 3D microstructures with high fluorescence emission which can be used in anti-counterfeiting applications. Besides, CDs may also be used as functional additives for 3D/4D printing of hydrogels and crosslinked resins to enhance the precision in printing and for enhancing photothermal stimulus-responsive behaviour. The combination of CDs & poly lactic acid in bioactive scaffolds, incorporated within Poly filaments, allows for the observation of bone regeneration because of their bright luminescence, leading to the possibility of real-time bioimaging of the healing process. Moreover, CDs have already demonstrated the photoinitiation capability & biocompatibility [115, 116] for ultrafast polymerization rates and high conversion of monomers, which are highly desirable for high-precision 3D printing of hydrogels. CDs impregnated in sodium polyacrylate inks for 3D printing also promise to make photoluminescent objects with low aggregation and, therefore yield high performance. Also, CDs have been used to improve the electrochemical properties of 3D-printed material [117, 118] by improving the ion mobility thereby enhancing capacitance, making them applicable in energy storage. Finally, CDs can enhance photocatalytic reactivity and charge separation efficiency for materials performing as TiO_2_ photoanodes [119, 120], which eventually becomes helpful in photoelectrochemical applications. In general, the strategy should be flexible to incorporate CDs within 3D printing materials for better functional properties, precision, and application of 3D-printed objects.

## CDs in formation of carbon dot nanocomposites (CDNC)

The interest of researchers is rapidly growing in field of CDPNCs due to their enhanced mechanical [121], thermal, and electrical properties [121], along with their potential for wide-ranging applications in fields like electronics [124], aerospace [123], and biomedical [122] engineering. Among CDPNCs, carbon quantum polymer nano composite dots (CQDPNCs) have shown remarkable optoelectronic properties like quantum confinement effects and band gap transitions which makes them highly preferable for biomedical applications like biosensors, bioimaging, and biomimetic implants. However, there is paradigm shift from CQDPNCs to bio-based CDPNCs synthesized from polysaccharides due to advantages like, (a) Biocompatibility, (b) Non-toxicity, (c) Economy, (d) Water Solubility and, (e) Functionalization. However, microwave pyrolysis is often used for synthesis of bio-based CDNCs which find potential applications in bioimaging  [125]. However, better quantum yield, enhanced stability, hydrophobicity and versatility in functionalization can be achieved by synthesizing CDs from Poly methacrylate (PMMA). In addition, incorporation of CDs in PMMA led to massive enhancement in dielectric properties; a further increase in relative permittivity can be obtained by doping CDs with neodymium to make them suitable for use in capacitors [126]. Apart from this application, functionalized CDNCs have also been found helpful in cross-linking hydrophilic polymers for achieving high mechanical strength in nanocomposite hydrogel. Despite numerous advantages, CDNCs generated from PMMA are hydrophobic due to which they cannot be used in aqueous environments [127]. Thus, this deficiency can be overcome by incorporation of polyurethane matrices which can enormously improve water solubility. In addition, dispersion efficiency [128], mechanical properties [129, 130] and thermal stability [131] of nanofillers like graphene nanoplatelets and carbon nanotubes was considerably improved by combining it with polyurethane incorporated CDs. Moreover, CDNCs having polyurethane offer excellent fluorescent properties but fluorescent CDNCs can be prepared from citric acid and urea and can be effectively used in vitro cell imaging where reaction conditions can tune their photoluminescence properties. Nevertheless, CDs with thermoplastic starch/ κ-carrageenan films have been found to imbibe antioxidant and UV-resistant properties exhibiting great potential in food packaging applications [132, 133]. Moreover, such CDNCs have been incorporated in hydrogels   [134–139]. Nevertheless, polystyrene sulfonate CDs composites have also showed improved electrical and optical properties for their use in flexible organic electronics. The chemically crosslinked CDs exhibit a pH-responsive fluorescence and have been employed in discriminating normal from cancerous cells in biomedical imaging. These different applications indicate that CD-based polymer nanocomposites widespread applications in various technological and biomedical applications. The synthesis of CDPNCs can be done using techniques like (a) Physical Mixing, (b) In situ Growth, (c) Solution blending, (d) Chemical grafting.

### Physical mixing

**Fig. 15:**
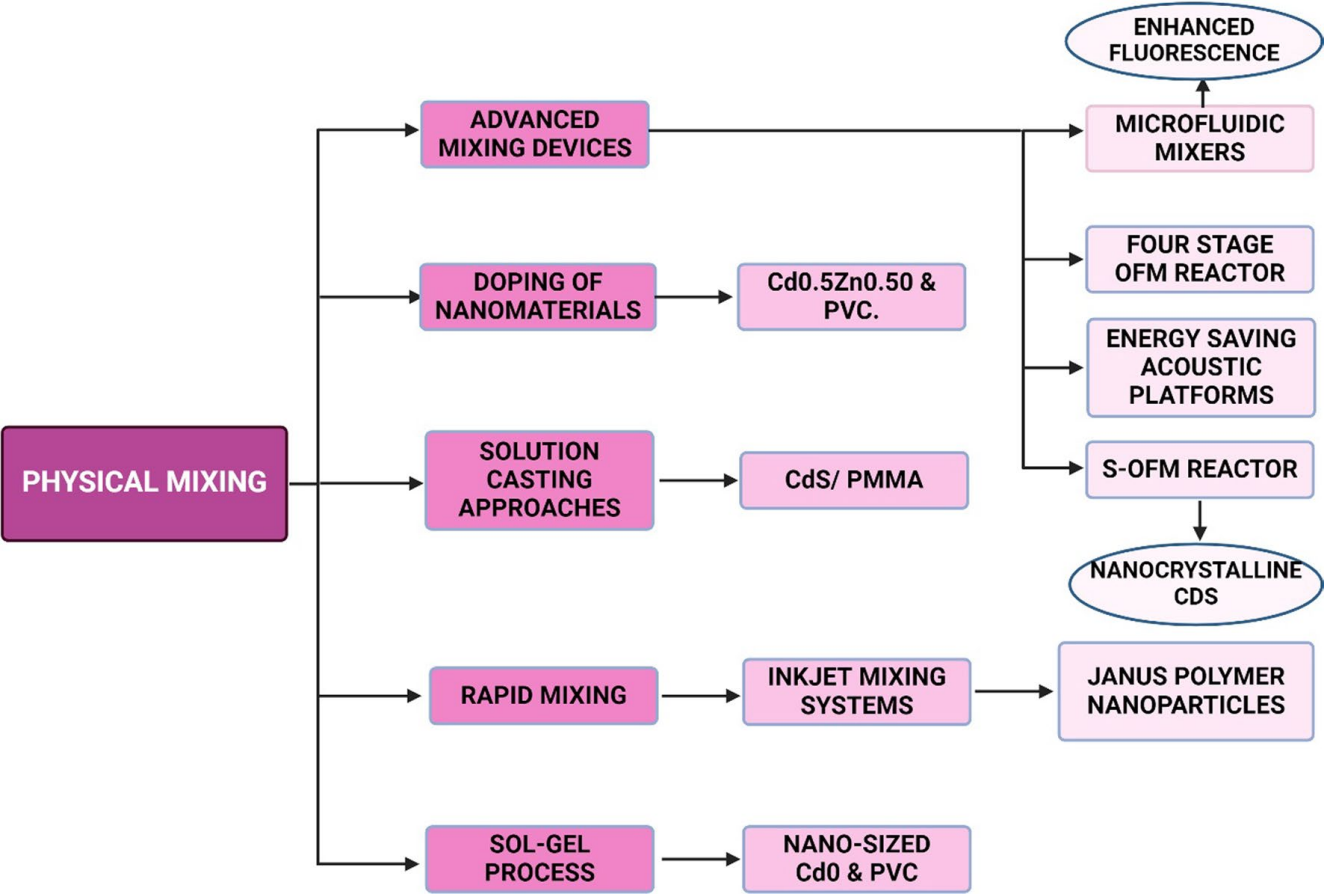
Physical Mixing method for generation of CDNC

Physical mixing is a simple and cost-effective method for the synthesis of CDNC which involves the mechanical blending of carbon-rich precursors without the need for complex chemical reactions (Fig. 15). The necessity of physical mixing arises from the simplicity of the process, as it allows for the production of CDNC without requiring specialized equipment or hazardous chemicals. This method is particularly advantageous in scenarios where resource availability is limited, and there is a need for rapid production of CDNC with minimal environmental impact. The mechanism underlying physical mixing typically involves the reduction in particle size through mechanical forces, such as grinding or milling [140–143], which facilitates the breakdown of larger carbonaceous materials into nanoscale particles. The resulting CDNC retain the essential properties of their precursors while acquiring the unique optical and electronic characteristics of nanoscale materials.

Despite its advantages, physical mixing as a synthesis method for CDNC has both pros and cons. On the positive side, it is an energy-efficient and environmentally friendly approach, avoiding the use of toxic reagents and harsh reaction conditions. Additionally, it allows for the easy scaling of CDNC production, making it suitable for industrial applications. CDNC produced through physical mixing can be employed in various fields, including bioimaging, and drug delivery, due to their biocompatibility and tuneable photoluminescence properties [144, 145]. However, the method also has limitations, such as the lack of precise control over the size and surface functionalities of the CDNC, which can affect their performance in specific applications. Moreover, the purity of the final product may be compromised, as physical mixing does not always ensure the complete removal of impurities or uniformity in particle size distribution. Therefore, while physical mixing offers a viable route for CDNC synthesis, it is often considered in combination with other methods to enhance the quality and functionality of the final nanomaterial.

The physical mixing process for the synthesis of carbon nanodots (CNDs) can be classified into several techniques, including ball milling, grinding, shearing, ultra sonification, and mechanical stirring. Ball milling involves high-energy collisions from milling media within a rotating cylinder, leading to the breakdown of precursor materials into nanoscale particles [146–149]. Grinding is a simpler process that uses manual or machine-assisted pulverization to achieve similar size reduction. Shearing applies shear forces, often in a liquid medium, to disrupt larger structures and form nanoscale CDs. Ultra sonication utilizes ultrasonic waves to induce cavitation in a liquid medium, creating high-energy bubbles that collapse and result in the formation of CDNC. Finally, mechanical stirring involves vigorous stirring in a liquid medium, dispersing and reducing the size of the precursor materials [150, 151]. Each of these techniques offers specific advantages and is suitable for different scales of production and applications, depending on the desired properties of the resulting CNDs.

Sol Gel method is one of the widely preferred methods of physical mixing technique used for CDNC synthesis. This method has been used for synthesizing CDNC by mixing Cadmium Oxide (CdO) with Poly Vinyl Chloride (PVC) and significant changes in dielectric properties were observed. Also, incorporation of Cadmium doped Zinc Oxide on PVC enhanced thermal stability and optical properties of nanocomposites. However, rapid mixing and inkjet systems are preferred over sol–gel methods for CDNC synthesis due to their ability to produce uniform particles with precise control over size and distribution, while also being faster and more scalable for industrial applications. This technique has been successfully used by authors on Janus polymers. Recently, advanced mixing devices, such as the oscillatory Baffled Reactor  enhances the mixing performance [152] and to control the synthesis of CDNC to achieve small and uniform size. Such strategies, together with microfluidic mixers, also help in reducing the size of nanoparticles to quite an extent, and they enhance fluorescence properties, which are very useful for bioimaging applications. Solution casting approaches have also been used to prepare CDNC with improved photocatalytic efficiency [153] [154] and viscoelastic properties. The results confirm that addition of CDs nanoparticles with Polymethyl Methacrylate (PMMA) increased the thermal stability and yielded non-agglomerated spherical particles dispersed within the polymer matrix. All these methods require strict control of mixing conditions to obtain the desired properties in CDNC.

### Solution blending

Solution blending is a technique that involves the dispersion of carbon precursors in a solvent [155–158], followed by various treatments to induce the formation of CDNC. The necessity of this process stems from its ability to produce CDNC with controlled sizes and surface functionalities, which are essential for specific applications. The mechanism typically involves dissolving or dispersing carbonaceous materials in a solvent, followed by sonication, heating, or chemical reduction to facilitate the formation of CDNC. This process allows for a more uniform distribution of CDs compared to physical mixing, leading to better control over their optical and electronic properties of CDNC. The choice of solvent and conditions during the blending process can significantly influence the size, surface chemistry, and overall quality of the CDNC, making this technique versatile for tailoring the properties of the nanodots for desired applications.

Solution blending offers several advantages, such as the ability to produce high-purity CDNC with tuneable properties, making them suitable for a wide range of applications, including bioimaging, drug delivery, and photocatalysis. It also allows for the functionalization of CDNC during synthesis, which can enhance their performance in specific applications. However, the process has its drawbacks, such as the potential toxicity of solvents used, the need for post-synthesis purification, and the potential difficulty in scaling up the process for industrial production. Solution blending can be classified based on the type of solvent used—such as aqueous or organic solvents—and the specific blending technique, whether it involves simple mixing, solvent evaporation, or more complex methods like electrospinning. Each classification offers distinct advantages and challenges, depending on the desired characteristics of the CDNC and their intended application. Solution blending of the CDs & PMMA nanocomposite films reduced the optical energy gap and increased electrical conductivity making them preferable for laser optics and photocatalysis applications [159, 160, 161]. Likewise, CDs-ZnS and CDs-TiO_2_ nanocomposites [162, 163] prepared using polyaniline via chemical precipitation showed excellent photocatalytic performance in dye degradation due to charge separation and higher light absorption. Solution Blending method increased thermal stability of CDNC [164–166[ but optical properties decreased considerably at higher filler concentrations. Nevertheless, incorporation of the Cd0.5Zn0.5O nanoparticles into a PVC matrix by a solution mixing process improved thermal stability [167], dielectric properties with decrease in optical energy band gap. Similarly, impregnation of C_d_T_e_ Quantum Dots in waterborne polyurethane (WPU) yielded transparent nanocomposite films which improved quantum yield and photochemical stability [168]. Furthermore, CDs nanoparticles stabilized with mercaptoethanol dispersed in poly vinyl alcohol matrix yielded excellent optical properties. Likewise, CDs nanoparticles embedded in a polyethylene glycol matrix exhibited high third-order optical nonlinearity making it preferable for photonic applications [169]. Furthermore, electrospinning techniques have been used to prepare light-emitting nanocomposite fibres, whereby CDs nanocrystals are embedded into polymer fibres. The prepared CdO nanocomposites have shown much enhancement in the photocatalytic activity under UV and natural sunlight, which provides remarkable increase in the dye decolorization rate. These studies reveal the versatility and superior properties of CDNC prepared by solution blending and confirm their potential in a broad spectrum of advanced applications [169–173].

### Chemical grafting

Chemical grafting is one of the widely adopted methods for synthesis of CDNC due to its ability to impregnate specific functional groups on surface of CDs thereby increasing the functional capacity of CDNC in several applications. The chemical grafting is mainly required to tune surface chemistry of CDs to enhance biocompatibility, solubility and interaction of CDs with different substrates. The chemical grafting mechanism involves impregnation of covalent molecules on CDs surface through esterification, click chemistry and amidation. These reactions are mainly triggered by reactive sites present on CDs surface like hydroxyl, carboxyl and amino groups which allow selective binding of grafting agents. Using chemical grafting process, surface properties of CDNC can be precisely controlled enabling their versatile application in different industries. The chemical grafting can also be subclassified into two categories namely, (a) Grafting to and (b) Grafting from process [174]. In the first method, the synthesized functional molecules are embedded into CDs surface which provides direct control over the property functionalization but, results in lower grafting densities. However, the second method involves in situ polymerization or direct growth of functional chains from CDs leading to higher grafting densities and robust surface. The chemically grafted CDNC have wide applications including drug delivery applications in which targeted interactions with the biological systems are facilitated by the CDs surface. Moreover, the functional groups can enhance sensitivity and selectivity of CDs in CDNC [175, 176]. However, it is difficult to achieve uniform properties of CDNC because of impurities and complexities involved in the process. Also, this process requires rigorous purification steps for removal of unreacted grafting agents and its by products which increases the synthesis time and overall cost. In spite of these shortcomings, chemical grafting is one of the most preferred methods of design of CDNC with customized functionalities for specific applications. Moreover, chemical grafting helps functionalize CDs with a large variety of organic or inorganic moieties thereby enhancing CDNC properties for specific applications. Specifically, this involves the covalent attachment of functional groups or molecules onto the surface of CDs through processes like esterification, amidation, or biocompatible chemical reactions which are simple to execute, high yielding, and have wide scope. CDNC properties can be further enhanced by grafting of polymers [177, 178], metal ions, or other nanomaterials into the CDs to modulate optical, electrical, and chemical properties of CDNC which makes them highly preferable for applications like bioimaging and sensors [179, 180]. Apart from the above-mentioned advantages, grafting process enhances the dispersion capability and stability of CDs in different media and confers new functionalities which improve CDs interaction with target molecules comprehensively enhancing CDNC properties. For example, polyethylene glycol grafting could improve the biocompatibility and circulation time of the CDNC within a biological system. Also, attachment of metal ions to CDs may enhance CDNC catalytic activity which is highly suited for environmental and energy applications. In summary, grafting offers a versatile and efficient strategy for tailoring CDNC with desired properties and functionalities for advanced technological applications. The researchers have observed grafting of CDs into polymer chains improves dissolution and compatibility of CDNC [181–183. Likewise, grafting of CdTe nanocrystals with PMMA through the condensation reaction (followed by free radical polymerization) resulted in more robust covalent bonding between the CdTe and PMMA segments enhancing thermal stability and optical properties of CDNC. Similarly, impregnation of CDs into poly or polystyrene using chemical grafting resulted in CDNC with controlled nanocrystal size with enhanced dispersion. CDNC produced by chemical grafting of ZnYCdO hybrid metal oxides embedded into carboxylate polyether sulfone were reported to had better functionality and improved dispersion. The overall synthesis of CDNC using chemical grafting is complex and involves advanced polymerization techniques but yields improved functional properties and adaptability of CDNC.

### In situ growth

In-situ growth is a powerful method for synthesizing CDNC [184, 185], where CDs are formed directly within a matrix material during the synthesis process. This technique ensures a uniform distribution of CDs within the matrix and often results in strong interactions between the CDs and the host material, leading to enhanced properties of the CDNC. The process begins with the selection of suitable carbon precursors, such as citric acid, glucose, or urea, which can decompose or carbonize under specific conditions to form CDs. These precursors are mixed with the matrix material or its precursors, which could be polymers, ceramics, metals, or other suitable materials to create CDNC. For carbon dot polymer nanocomposites (CDPNC), the carbon precursor is mixed with monomers before the polymerization process [186–189. Thereafter, in-situ growth of CDs is initiated by applying specific conditions such as heat, pressure, microwave irradiation, or hydrothermal treatment. During this process, the carbon precursors decompose or react to form CDs, which are simultaneously integrated into the host matrix to create CDNC, preventing aggregation and ensuring uniform dispersion of CDs.

As the CDs grow, they become embedded within the matrix, forming the CDNC. The matrix material may simultaneously polymerize, crystallize, or solidify, locking the CDs into place. This in-situ approach offers several advantages, including uniform dispersion of CDs within the matrix, strong interfacial bonding, and enhanced properties such as improved optical performance, mechanical strength, and thermal stability. The method is versatile, adaptable to various matrix materials and synthesis conditions, and suitable for a wide range of applications. For instance, in-situ grown CDNC are widely used in optoelectronics due to excellent photoluminescence which makes them ideal for LEDs, solar cells, and other devices. They are also used in sensing applications, where the enhanced optical properties of the CDNC enable the detection of specific molecules or environmental conditions, and in biomedical fields, where their biocompatibility and versatility make them useful in drug delivery systems, bioimaging, and tissue engineering. Additionally, these CDNC are employed in energy storage devices like supercapacitors and batteries, where the CDs improve electrical conductivity and energy storage capacity.

**Fig. 16:**
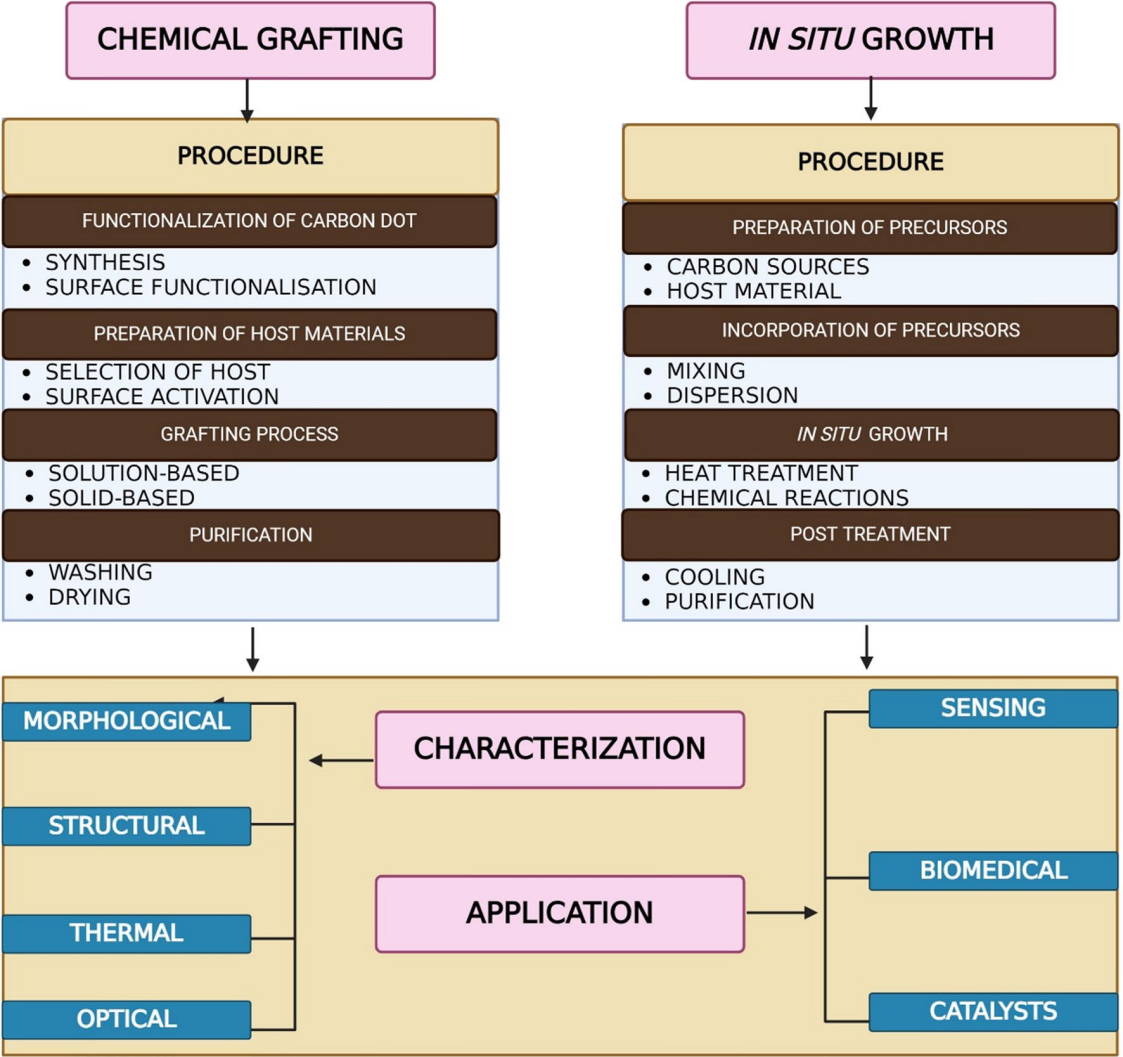
Comparison between chemical grafting and in situ growth for generation of CDNC

For example, in the in-situ growth of CDs in a polyurethane matrix, a carbon precursor like citric acid is mixed with the polyol component of polyurethane. When the isocyanate component is added, polymerization is initiated, and the mixture is heated to induce carbonization of the citric acid, forming CDs within the growing polyurethane matrix. The resulting material is a polyurethane-CDNC with uniformly dispersed CDs, exhibiting enhanced mechanical properties and fluorescence. Overall, in-situ growth is a highly effective method for synthesizing CDNC, offering significant benefits in terms of uniformity, integration, and enhanced functional properties across various fields. The comparison between chemical grafting and in situ growth is shown in Fig. 16 and detailed comparison of different synthesis methods of CDNC is shown in Table 7.

**Table 7:** Detailed comparison of physical mixing, solution blending, in situ growth and chemical grafting for synthesis of CDNC

Aspect	Physical mixing	Solution blending	Chemical grafting	In situ growth
Definition	Simple physical mixing of carbon dots with the host material	Dissolution of carbon dots and host material in a common solvent, followed by solvent evaporation	Covalent attachment of carbon dots to the host material	Formation of carbon dots within the host material matrix
Process Complexity	Low	Moderate	High	High
Interaction Type	Physical	Physical	Chemical (Covalent Bonding)	Physical and Chemical
Equipment Required	Basic mixing equipment	Stirring or ultrasonication equipment, evaporator	Chemical reactors, functionalization agents	Autoclave or microwave reactor for hydrothermal/solvothermal processes
Time consumption	Low	Moderate	High	High
Temperature Requirements	Ambient or mild heating	Ambient or mild heating	May require elevated temperatures for grafting reaction	Elevated temperatures for hydrothermal/solvothermal processes
Dispersion Quality	Poor to moderate	Good	Excellent	Excellent
Uniformity of Nanocomposite	Variable, often non-uniform	Generally good, dependent on mixing efficiency	Very uniform due to strong bonding	Very uniform, depends on growth conditions
Stability of Composite	Low to Moderate	Moderate	High	High
Strength of Interaction	Weak physical forces	Weak physical forces	Strong covalent bonds	Strong chemical and physical interactions
Applications	Simple applications, low-cost products	Coatings, films, flexible substrates	High-performance materials, where strong interactions are needed	Advanced applications requiring precise control of nanostructures
Advantages	Simple, low initial cost, easy scalability	Better dispersion than physical mixing, relatively simple process	Strong interaction, excellent stability, high performance	Uniform distribution, high interaction, tailored properties
Disadvantages	Poor dispersion, non-uniform properties	Requires solvent removal, possible solvent residues	Complex, time-consuming, requires precise control of reaction conditions	Complex, requires high temperature/pressure, challenging to control particle size and distribution
Examples of Use	Basic composite materials, bulk products	Functional films, coatings, flexible electronics	Advanced catalysis, high-performance sensors, durable coatings	Photocatalysis, energy storage materials, biomedical applications
Environmental Impact	Low, depends on the mixing method used	Moderate, depends on solvent used	Variable, can involve toxic reagents and by-products	Moderate to high, depends on synthesis conditions and precursors used

### Carbon dot-based metal nanocomposite (CDMNC)

Recently, CDMNC have drawn intensive interest due to their unique properties that find versatile applications in various fields [190]. CDMNC have been demonstrated as the next generation of tuneable luminescence materials with good conductivity and biocompatibility which can be prepared or synthesized using different synthetic approaches, including hydrothermal processing or pyrolysis of metal organic frameworks. In addition, these nanocomposites yield better catalytic performance [191], especially in electrochemical reactions, such as the reduction reaction of oxygen with good catalytic activity and stability due doping of carbon composites embedded in metals like Cu, Co, Mn, and Fe. CDs have C–C or C–O bonds, which have been functionalized through plasmonic metal nanoparticles (NPs), both silver and gold, to enhance fluorescence, thus rendering them useful for sensing and bioimaging application. Furthermore, CDMNC created by incorporating metals such as Bi, Cd, and Ti onto CDs matrix to enhance the photocatalytic hydrogen yield due to the charge separation and electron transfer properties. CDs also can be used as reducing and stabilizing agents in the green synthesis of noble metal nanoparticles [192–194], such as Ag and Au, and potential applications in the environmentally friendly production of nanomaterials. CDMNC form a very good option for multifunctionality 3D printing as CDs could be considered an essential precursor in developing functional additives that would enhance the lay accuracy of the print, enhanced photoluminescence and increased electrical conductivity. Further, CDMNC represent a great scope for their antibacterial activity since they can efficiently inactivate bacteria upon sunlight irradiation of TiO_2_ nanocomposites. Moreover, functions and applications of CDMNC are very diversified and promising for further technological development in catalysis, sensing [175], and biomedicine [195, 196].

### Carbon dot based metal oxide nanocomposite (CDMON)

**Fig. 17:**
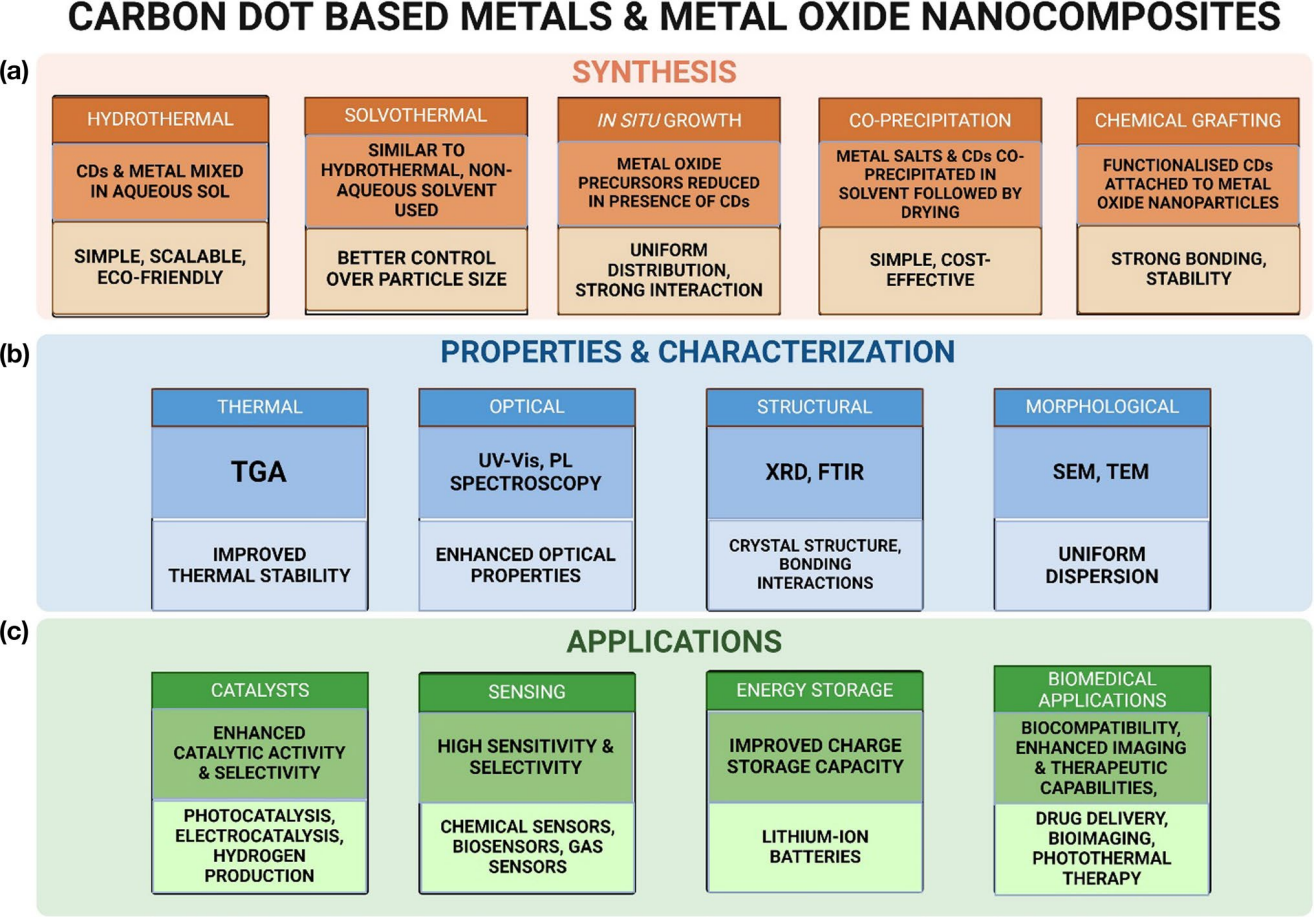
Comparison of **a** synthesis methods, **b** properties and characterization, **c** applications for CDMNC and CDMON

Metal oxide nanocomposites based on CDs find application in numerous domains due to their unique characteristics [196, 197]. CDMON uses CDs which are well-established as a class with tuneable photoluminescence, high conductivity, and biocompatibility; they are synthesizable using several routes, including hydrothermal processes and pyrolysis of metal–organic frameworks (MOFs). CDMON emerged with high catalytic performances, particularly in the electrochemical reactions of oxygen reduction reaction (ORR) and oxygen evolution reaction (OER). This high catalytic performance is also exhibited by all embedded metal nanoparticles, including Co, Cu, Mn, and Fe in Nitrogen-doped carbon composites. Moreover, CDs can be functionalized with plasmonic metal nanoparticles (silver and gold) to customize their fluorescence properties for more sensitive sensing and bio-imaging applications. Also, better photocatalytic hydrogen production can be obtained by doping the CDs with metals like Bi, Cd, and Ti. This is attributed to due to better charge separation and electron transfer properties. In addition, CDs can be efficiently used for green synthesis of metal nano particles since it does not require any stabilizing agents. Moreover, CDs can be used as photocatalysts to design functional additives in hydrogel and resin. Hence, it can render enhanced accuracy and stimuli-responsive properties in 3D/4D printing of hydrogels and resins. Also, CD-based nanocomposites have been very helpful in bacterial applications, especially in the photocatalytic deactivation of bacteria under sunlight, aquaculture and other biological systems. CDMON have diversified functionalities and applications which acknowledge the importance of this development in material science and technology. CDs significantly enhance the properties of CDMON, including improved dispersion, optical characteristics, and elevated catalytic activity. Incorporation of CDs into metal oxides nanocomposites exhibits significantly enhanced fluorescence, due to covalent bonding of CDs with plasmonic metal nanoparticles, such as Ag or Au they’re by enhancing the mechanical and electric properties of CDMON. A significant improvement in the tensile strength was observed for CDMON with copper matrix reinforced with carbonized polymer dot possessing a high electrical conductivity of 97.2% & 20% & higher tensile strength in comparison to pure copper [198]. Conversely, CDs can potentially enhance the catalytic properties of CDMON [207]. The functionalization of CDs with metal ions like manganese has significantly improved their enzymatic activities, thus forming efficient enzymes for various applications, including sensitive ascorbic acid detection. CDs further enhanced the quality of dispersion resulting in increase of mechanical properties and thermal stability of polyurethane composites (with graphene nanoplatelets and carbon nanotubes dispersed) in carbon quantum dots. In addition, a significant improvement in properties of CDMON were observed by using CDs prepared from renewable biomass and incorporated with metal oxides (titanium dioxide and iron oxide). Likewise, exceptional electrical conductivity and stability was observed due to mixing of CDs with MXene showing promising applications in energy-related aspects, such as an electrocatalyst of fuel cells and electrode material for batteries. Generally, these distinct parts of CDs, including high solubility, low cytotoxicity, and coordination towards metals, make them very useful in the performance improvement of CDMON. The comparison of (a) Synthesis methods, (b) properties and characterization, (c) Applications of CDMNC and CDMON are shown in Fig. 17.

## Problems encountered with possible solutions in the use of CDs in different industrial applications

The details of the different aspects related to carbon dot nanocomposite has been depicted in Fig. 7 and Table 8. Although CDNC have widespread applications but there are some challenges as discussed below.

- *Synthesis challenges* The main challenges for the application of CDNC are the synthesis processes. There is an assumption of uniform size and high quantum yield that is unlikely, given that there are different synthesis methods, such as chemical oxidation, microwave, and hydrothermal synthesis. Consequently, this often results in CDNC with non-uniform properties presenting a challenge of mass production. Therefore, methods and standards for synthesis of CDNC should be uniform by adopting perfect synthetic methodologies using controlled precursors and reaction conditions, which provide for the synthesis of CDNC with reproducible properties along with improvement of post-synthesis purification.
- *Toxicity concerns* The primary concern with the application of CDNC, particularly in biomedicine, is related to the materials that can bring about toxicity. The heavy metals or other toxic components and other media used in the synthesis process may be cytotoxic. This limitation might be overcome by using biocompatible and environment-friendly precursors. Also, CDNC biocompatibility could be engineered through surface modification and functionalization with biocompatible polymers or molecules. However, several applications may require comprehensive toxicity studies before recommendation of CDNC.
- *Limited understanding of mechanisms* Therefore, despite the intensive study of CDNC, the actual mechanisms for determining the optical and electronic properties are still quite elusive. Consequently, the difficulty lies in the tuning of CDNC to become optimal materials for sensors or bioimaging applications. This necessitates a detailed investigation of the fundamental properties of CDNC using advanced characterization techniques like spectroscopy and microscopy. A combined effort and collaboration of chemists, physicists, and material scientists are needed for development of CDNC with tuneable properties.
- *Stability issues* One of the main challenges experienced by CDNC operation by photobleaching under the constant exposure of light especially in extreme conditions. This limits CDMC application in biological devices used for longer time duration. The stability is mainly based on core shell structures and surface passivation assembled by using solid CDNC. Further, stabilizing agents can be used to prevent this degradation. Nevertheless, continuous testing should be employed to ensure long time durability under varying environmental conditions.
- *Scalability of production* Scalability is one of the major issues associated with CDNC as mass production of CDNC needs to be synchronized with consistency and quality delivery. The variation in different product batches and production cost or some of other hurdles faced in commercialization of CDNC. Therefore, scalability and aspect of cost economics should be considered special parameter. The green synthesis method utilizing natural precursors an eco-friendly process can reduce the cost and help in mass scalability. Moreover, continuous flow synthesis and automation of the flow process are critical parameters for ensuring consistent mass production.
- *Application-Specific Optimization* In case of CDNC designed for specific purpose. The properties of such CDNC like size, Surface, chemistry and Photo Luminance needs to be optimized. This process is quite tedious and requires a detailed optimization. Therefore, and exhaustive database on property-structure relationship needs to be formulated for efficient optimization. The utilization of machine learning algorithms with clearly defined constraints and boundary conditions can prove to be quite useful in optimization.

**Table 8:** CDs metal and metal oxide nanocomposite

Property/aspect	Carbon dot-metal nanocomposites	Carbon dot-metal oxide nanocomposites
Composition	Combination of carbon dots with metals (e.g., Au, Ag, Pt)	Combination of carbon dots with metal oxides (e.g., TiO_2_, ZnO, Fe_3_O_4_)
Synthesis Methods	Chemical reduction, electrochemical deposition, hydrothermal methods	Sol–gel method, hydrothermal synthesis, chemical vapor deposition
Structural Characteristics	Highly crystalline metallic particles & Amorphous particles embedded in carbon dots	Semi-crystalline or amorphous metal oxide phase with carbon dots
Optical Properties	Strong surface plasmon resonance, tunable emission based on metal	Strong absorption in UV–Vis, enhanced photocatalytic properties
Electrical Properties	Enhanced electrical conductivity due to metal presence	Moderate electrical conductivity, dependent on the type of metal oxide
Catalytic Activity	Highly efficient as electrocatalysts, photocatalysts	High photocatalytic efficiency under UV/visible light
Stability	Moderate stability depending on the environmental conditions	Higher stability due to the robust nature of metal oxides
Applications	Sensors, electrocatalysis, photothermal therapy, bioimaging	Water treatment, environmental remediation, energy storage, sensors

Table 9 summarizes the challenges associated and possible potential solutions.

**Table 9:** Challenges associated and suggested solutions for CDs

Problem	Description	Solution
Aggregation of Carbon Dots	Carbon dots tend to aggregate, leading to reduced surface area and diminished properties	Use surface functionalization to introduce repulsive forces between carbon dots, thereby preventing aggregation
Poor Dispersion in Host Matrix	Uneven dispersion can lead to non-uniform properties and reduced performance of the nanocomposite	Employ ultrasonication, high-shear mixing, or chemical grafting to ensure even dispersion of carbon dots within the host matrix
Weak Interaction with Host Material	Weak interactions between carbon dots and the host material can result in poor mechanical and thermal properties	Utilize chemical grafting or in situ growth techniques to form strong covalent bonds between carbon dots and the host material
Limited Stability and Durability	Carbon dot nanocomposites may degrade over time, especially under harsh environmental conditions	Apply surface passivation and encapsulation techniques to enhance the stability and durability of carbon dots within the nanocomposite
Toxicity and Biocompatibility Issues	Some carbon dots may exhibit cytotoxicity, limiting their use in biomedical applications	Synthesize carbon dots from biocompatible precursors and thoroughly purify them to remove toxic by-products. Conduct comprehensive biocompatibility testing
Scale-Up Challenges	Difficulties in scaling up the synthesis process while maintaining uniform properties can hinder commercial application	Develop scalable synthesis methods, such as continuous flow processes, and optimize reaction conditions for large-scale production without compromising quality
Cost of Production	High production costs can limit the widespread adoption of carbon dot nanocomposites in various industries	Optimize synthesis routes to use cost-effective raw materials and energy-efficient processes. Explore recycling and reuse of materials to reduce overall costs
Optical Property Control	Achieving consistent and tunable optical properties can be challenging due to variations in size and surface chemistry of carbon dots	Fine-tune synthesis parameters, such as temperature, time, and precursor concentration, to control the size and surface chemistry of carbon dots
Environmental Impact	The environmental impact of the production and disposal of carbon dot nanocomposites is not fully understood	Conduct life cycle assessments and develop environmentally friendly synthesis and disposal methods. Promote the use of renewable and non-toxic materials
Compatibility with Existing Systems	Integrating carbon dot nanocomposites with existing systems and technologies can pose challenges	Design nanocomposites with properties tailored for compatibility with existing systems. Collaborate with industry stakeholders to facilitate seamless integration

## Specific research gaps and proposing potential areas for future investigation

### Challenges in green synthesis protocols

#### Lack of standardization

Current green synthesis methods for CDs vary widely in terms of the precursors, synthesis conditions, and post-synthesis treatments. This variability leads to inconsistencies in the physicochemical properties of CDs, affecting the reproducibility of results and limiting scalability for industrial applications.

#### Incomplete understanding of formation mechanisms

A thorough knowledge and understanding of mechanisms governing growth and nucleation of CDs through green synthesis methods is missing. Without comprehensive knowledge of these mechanisms, it is quite difficult to fine tune and control the properties of CDs for different applications. Moreover, future research should focus on synthesis parameters affecting optical and structural properties of CDs.

#### Need for scalable and reproducible protocols

The consistent quality and green synthesis, scalable protocols and process standardization but essential for ensuring quality of CDs in case of mass production. Therefore, elaborate study is required for design of efficient green synthesis process.

#### Understanding interactions in nanocomposites

In spite of CDs application in diverse fields still, the interaction between CDs and host matrix have not been subjected to a detailed study as evident from the previous research works. This understanding is very critical for optimizing the performance of CDs in different applications like environmental remediation, energy storage and biomedicine.

#### Synergistic effects between CDs and matrix materials

A comprehensive understanding of influence of CDs on catalytic properties of composites you said required to formulate the potent pollution control strategies. A clear understanding of these effects can improve therapeutic and biocompatibility efficacy of CDs.

#### Long-term stability and environmental impact

Long term stability of CDs especially in mass applications have not been explored so far. The potential degeneration of nanocomposites along with environmental impacts have not been thoroughly studied. Future research areas pertaining to CDs should give more priority to development of stable and durable CDs. Moreover, life cycle assessment of CDs should be carried out to determine the long-term effects.

## Future directions for research on CDs

The future of green-synthesized CDs is full of opportunities, but certain critical research questions need to be addressed to harness potential of CDs. Detailed research into the core mechanisms responsible for green synthesis methods is one area that needs further attention. However, most of these methods are considered to be eco-friendly, but the detailed biochemical and physicochemical pathways actually driving CDs formation remain poorly understood in various green syntheses methods. Future studies should focus towards, definitive mechanism elucidation—possibly by in situ spectroscopy and high-resolution microscopy-based advanced analysis associated with syntheses to optimize conditions for enhanced quality and consistency of resultant CDs. Scalability is another main challenge in green syntheses methods with laboratory scale experimentation showing feasibility of green synthesis methods for CDs but the industry scale adaptation is quite difficult. Therefore, future research should be focused on making green synthesis methods cost effective and easy to implement in mass production of CDs. Therefore, scalability of this methods should consider optimum conditions of reaction in terms of temperature, pressure, and concentration of reactants, so as to allow better yields and reproducibility. Nevertheless, the collaboration between the industry and academia is making it easy to transfer these kinds of technologies from the research laboratory to commercial production so that they are able to work effectively in real applications.

Moreover, the standardisation of green synthesis protocols there is no standard procedure in the preparation and characterization of CDs. In this direction, there lies an instant need for the scientific community to come up with a universally acceptable guideline when adopting a green synthesis or other specific methodologies, precursor selection, appropriate conditions of synthesis, and characterization tools to be used. Such standards will increase the reliability of results obtained from research and help in regulatory approval, eventually paving the way for wide adoption of these materials in their respective industries.

While CDs have enormous potential, it is emerging that there is a significant need to optimize them for desired uses. Future CDs research has to be tailored to their size, surface functionalization, and photoluminescence properties so that they meet the target applications' prerequisites. For example, in biomedical applications, there is a dire need for the construction of CDs with enhanced biocompatibility and targeted drug delivery features. Similarly, in the case of environmental remediation, adsorption capacity and selectivity of CDs towards pollutants has to be improved. The multifunctionality of CDs can only come to the fore when application-specific optimization takes place. Finally, environmental and economic impacts of green-synthesized CDs should be worked out for their sustainable development. Therefore, the in-depth life cycle assessment should be a main element of evaluation in future research studies of such kind of materials, since it has an interaction impact on the environment at each stage—from synthesis to disposal. Moreover, economic feasibility studies on the comparison of cost-effectiveness with traditional approaches are to be put in place for green synthesis methodologies. Bottleneck identification and solution proposition will be, therefore, indispensable for commercial viability and environmental sustainability through the use of renewable raw materials and energy-efficient processes in making green-synthesized CDs applicable at large.

As the field of CDs continues to evolve, several key areas require focused research to address existing challenges and unlock the full potential of these materials.

### Development of standardized green synthesis protocols

#### Potential solutions

Conduct comparative studies to evaluate the effects of various natural precursors and synthesis conditions on the size, morphology, and surface chemistry of CDs.

Develop and validate a set of standardized synthesis protocols that can be universally applied, ensuring reproducibility and scalability.

Investigate the role of key parameters such as pH, temperature, and reaction time in the formation of CDs, aiming to establish optimal conditions for specific applications.

### Understanding the mechanisms of CDs formation

#### Potential solutions

Use advanced characterization techniques, such as in situ spectroscopy and electron microscopy, to monitor the formation of CDs in real-time.

Develop theoretical models to predict the influence of different synthesis conditions on the formation and properties of CDs.

Investigate the role of natural precursors’ molecular structures in dictating the properties of the resulting CDs, with the aim of guiding the selection of precursors for targeted applications.

### Elucidating synergistic effects in nanocomposites

#### Potential solutions

Conduct systematic studies on the interfacial interactions between CDs and different matrix materials, focusing on how these interactions influence the mechanical, optical, and electronic properties of the nanocomposites.

Explore the use of surface functionalization techniques to enhance the compatibility and bonding between CDs and matrix materials, thereby improving the overall performance of the composites.

Investigate the potential for creating hybrid nanocomposites that combine CDs with other nanomaterials, such as metal oxides or polymers, to achieve synergistic effects that enhance their multifunctionality.

### Long-term stability and environmental impact assessment

#### Potential solutions

Perform accelerated aging studies to evaluate the long-term stability of CDs under different environmental conditions, such as exposure to UV light, moisture, and extreme temperatures.

Develop life cycle assessment models to evaluate the environmental footprint of CDs, from synthesis to disposal, including the potential release of degradation products.

Investigate the biodegradability and toxicity of CDs and their degradation products, with the aim of designing nanocomposites that are both high-performing and environmentally benign.

### Exploring new applications and functionalities

#### Potential solutions

Explore the use of CDs in emerging fields such as flexible electronics, wearable sensors, and energy-harvesting devices, where their unique optical and electronic properties could be particularly beneficial.

Investigate the potential for using CDs in advanced biomedical applications, such as targeted drug delivery, bioimaging, and photothermal therapy, where their biocompatibility and tuneable properties could offer significant advantages.

Develop multifunctional CDs that can simultaneously perform multiple tasks, such as pollutant detection and degradation, or energy storage and conversion, by exploiting the diverse properties of carbon dots.

By pursuing the proposed solutions, the field of CDs can advance toward creating more reliable, effective, and sustainable materials. These advancements will not only enhance the current applications of CDs but also open up new avenues for their use in addressing some of the most pressing challenges in technology, healthcare, and environmental sustainability.

## General engineering solutions (GES) for resolving issues related to CDs

The general engineering solutions for resolving issues related to CDs have been shown in Table 10 and discussed in detail below.

**Table 10:** General engineering solutions for problems associated with CDs

Problem	Associated issue	Engineering solution
Limited scalability of synthesis	Lab-scale synthesis methods are not easily scalable for industrial production	Develop scalable green synthesis methods such as microwave-assisted or hydrothermal synthesis
Fluorescence quenching	Reduction in fluorescence intensity due to external factors	Enhance surface passivation techniques or use dopants to maintain fluorescence
Low quantum yield	Poor light emission efficiency compared to other quantum dots	Optimize the synthesis process or add surface passivation layers to enhance quantum yield
Toxicity concerns in biological applications	Potential cytotoxicity of CDs when used in vivo	Ensure proper surface functionalization with biocompatible molecules and conduct thorough toxicity studies
Aggregation in aqueous solutions	Carbon dots tend to form aggregates in water, reducing performance	Use of stabilizing agents or surface modifications to prevent aggregation
Limited stability under harsh conditions	Degradation of CDs under extreme pH, temperature, or UV exposure	Improve surface coating or functionalization to protect CDs from degradation
Difficulty in surface functionalization	Surface functionalization may not be uniform or reproducible	Develop robust chemical modification protocols for consistent functionalization
Environmental concerns regarding large-scale production	Use of non-green synthesis methods can lead to environmental pollution	Focus on green chemistry approaches using biomass or environmentally friendly precursors

### Scalability of synthesis methods

*Problem* CDs synthesis is typically done on a small, laboratory scale, which limits industrial applications, especially for large-scale manufacturing. The solution is as follows

- *Microwave-Assisted Synthesis*: This technique offers rapid heating and uniform energy distribution, which enables mass production of CDs with consistent quality. Additionally, it uses less energy and shorter reaction times, making it environmentally friendly.
- *Hydrothermal and Solvothermal Synthesis*: These scalable methods allow for the use of green solvents and renewable precursors, making them attractive for large-scale applications. Engineers can design high-throughput reactors for continuous production, enabling integration into manufacturing sectors that require bulk material processing, such as electronics and textiles.
- *Biomass-Based Synthesis*: The development of carbon dots from biomass (e.g., food waste, agricultural by-products) provides an environmentally sustainable solution for industries that need to meet green manufacturing goals, such as automotive, aerospace, and materials science. Engineers could automate processes to convert biomass to CDs in large quantities, reducing overall costs.

### Fluorescence quenching

*Problem*: CDs often experience fluorescence quenching in practical applications, which limits their effectiveness in bioimaging, sensors, and other photonic technologies. The solution is as follows.

- *Surface Passivation*: Surface passivation through encapsulation in biocompatible polymers (like PEGylation) or through doping (e.g., nitrogen or sulfur doping) can help stabilize the fluorescence properties of CDs. Engineers could focus on developing more efficient surface coatings that maintain the optical properties of CDs in harsh conditions, such as within the human body for medical diagnostics.
- *Use of Dopants*: Incorporating metallic elements (e.g., nitrogen, sulphur, or phosphorous) during synthesis enhances the optical properties of CDs by preventing fluorescence quenching. These doped CDs could be integrated into light-emitting diodes (LEDs) or solar cells, where stable fluorescence is critical for efficiency.

### Low quantum yield

*Problem*: CDs generally have a lower quantum yield compared to other quantum dots (QDs), limiting their efficiency in applications such as displays, sensors, and energy devices. The solution is as follows.

- *Doping with Metals or Heteroatoms*: Engineering CDs with metallic nanoparticles or heteroatoms (e.g., sulphur, nitrogen) significantly improves quantum yield by introducing new electronic states that enhance light absorption and emission. This approach is useful in developing high-efficiency light-based devices (like LED screens and laser diodes).
- *Surface Passivation and Core–Shell Structures*: Developing core–shell nanostructures where the CD core is coated with a protective shell (such as silica) can prevent non-radiative recombination of excitons, thus boosting the quantum yield. Engineers working in optoelectronics and photovoltaics can leverage this technology to create brighter and more efficient devices.

### Biocompatibility for biomedical applications

*Problem*: CDs may exhibit cytotoxicity, especially when used for in vivo applications like drug delivery or imaging. The solution is as follows.

- *Surface Functionalization with Biocompatible Molecules*: Engineers could improve biocompatibility by attaching hydrophilic groups, such as carboxyl, amine, or hydroxyl groups, to the surface of CDs. These modifications enable better interaction with biological tissues and prevent immune responses. For instance, in drug delivery systems, CDs can be functionalized with targeting ligands to enhance their specificity toward cancer cells while reducing off-target toxicity.
- *Thorough Toxicity Evaluation*: Conducting comprehensive cytotoxicity and hemocompatibility studies is essential before deploying CDs in medical devices or drug delivery. Engineers should focus on creating biocompatible coatings that degrade safely within biological environments, ensuring CDs do not accumulate in organs or tissues.

### Aggregation in aqueous solutions

*Problem*: CDs tend to aggregate in water, which hampers their effectiveness in applications like environmental monitoring and drug delivery. The solution is as follows.

- *Use of Stabilizing Agents*: Engineers can introduce surfactants, polymers, or functional groups that prevent aggregation by improving the colloidal stability of CDs in aqueous solutions. For instance, in water treatment systems, CDs must remain well-dispersed to efficiently degrade pollutants.
- *Surface Modification with Hydrophilic Groups*: Modifying the surface of CDs with hydrophilic functional groups (like –OH or –COOH) helps maintain their dispersion in aqueous environments. This is particularly relevant for environmental applications where CDs must remain stable in water for pollutant detection or degradation.

### Limited stability under harsh conditions

*Problem*: CDs can degrade or lose functionality under extreme pH, UV radiation, or high temperatures, which affects their performance in outdoor and industrial environments. The solution is as follows.

- *Protective Coatings*: Coating CDs with robust, chemically inert layers such as silica, polymers, or metal oxides can protect them from environmental stressors. Engineers developing photocatalysts for wastewater treatment, for instance, can use such coatings to ensure long-term durability.
- *Hybrid Composites*: Combining CDs with metal oxides (such as TiO_2_) or other semiconductor materials can improve their thermal and UV stability. These hybrid nanocomposites are useful in applications like solar cells and water purification systems, where materials are exposed to harsh conditions.

### Environmental impact of large-scale production

*Problem*: Non-green synthesis methods used for large-scale production of CDs could result in environmental degradation, especially when using toxic reagents or solvents. The solution is as follows.

- *Green Chemistry Approaches*: Engineers can adopt green chemistry principles, using natural precursors like agricultural waste, plant extracts, or food waste. Additionally, environmentally friendly solvents and low-energy processes (like microwave synthesis) could be integrated into the production pipeline. This shift is particularly beneficial for the manufacturing sector, where sustainability is a growing concern.
- *Sustainable Manufacturing Practices*: Incorporating waste minimization, recycling, and energy-efficient processes ensures that the large-scale production of CDs aligns with industrial sustainability goals. This includes closed-loop systems where solvents and other reagents are recycled to reduce waste.

### Applications-based differentiation

- *Environmental Remediation*: CDs can serve as photocatalysts in degrading pollutants in water and air. Engineers focusing on environmental engineering can optimize CDs by creating composites with other nanomaterials to improve the degradation rate of pollutants like dyes, pesticides, or heavy metals.
- *Biomedical Applications*: Surface-engineered CDs can be utilized in bioimaging, drug delivery, and photothermal therapy. In these applications, the engineering solution involves modifying CDs to be non-toxic, stable, and biocompatible for safe interaction with biological systems.
- *Energy Devices*: In energy storage and conversion systems, such as solar cells or supercapacitors, CDs serve as electron mediators. Engineers can enhance these properties by developing hybrid materials, where CDs are combined with other semiconductors or conductive polymers to increase efficiency.
- *Optoelectronics*: For applications like light-emitting diodes (LEDs), the stability and quantum yield of CDs are critical. Engineering solutions focus on passivation techniques and heteroatom doping to enhance light emission properties, making them suitable for the next generation of display technologies.

## Conclusions

A vast amount of research work has been conducted in the process of green synthesis and application of CDs. However, several research groups and challengers need to be addressed to fully utilize potential of CDs. The non-existence of standardized protocols on green synthesis of CDs induces inconsistencies in their physiochemical properties which effects scalability and reproducibility of these materials especially for industrial applications. Moreover, the mechanism for synthesis of CDs has not been fully understood which limits the ability to optimize and control CDs properties for specific usage. Therefore, these issues need to be addressed through systematic research to make significant advancement in this field necessary for the production of CDs with desired properties at consistent scale.

In addition to synthesis methods, CDs performance is intrinsically linked to the interactions between metrics and CDs. This this aspect has not been fully explored especially in complex real-world environment. Moreover, an elaborate understanding of synergistic effect is very essential for optimizing CDs for applications like environmental remediation, energy storage and biomedical engineering. In environmental remediation, a detailed knowledge of influence of CDs on catalytic properties of composites is very critical in formulation of sustainable control mechanisms.

In biomedical field, the detailed insight into interaction between CDs and catalytic properties of nanocomposites is enhancing therapeutic and biocompatibility efficacy of CDs making them suitable for clinical applications. Moreover, there is an urgent necessity for investigation of degradation, environmental impact a long-term stability especially in scaling their laboratory usage to industrial applicability. A deep understanding of these aspects will prevent CDs in harming ecology and environment.

The inventions required to address these challenges represent a transformative shift in design, synthesis and application of CDs.

Developing scalable, reproducible green synthesis methods will lay the groundwork for the widespread adoption of CDs across various industries. Concurrently, research focused on elucidating the interaction dynamics within CDs will unlock new functionalities and applications, further driving the evolution of these materials. By addressing on a future stability and environmental impact of CDs, it can be ensured that their benefits are sustainable and do not come at the cost of environmental or human health. Ultimately, the successful resolution of these challenges will elevate CDs from a promising research topic to a cornerstone of next-generation multifunctional nanomaterials, driving innovation, sustainability, and practical solutions in a wide range of fields, from energy to healthcare. This robust foundation will not only enhance the performance and applicability of CDs but also position them as key players in the global effort to create a more sustainable and technologically advanced future.

## Data Availability

No datasets were generated or analysed during the current study.
